# Chicken-origin Cluster 3.2 Tembusu virus exhibits higher infectivity than duck-origin Cluster 2 Tembusu virus in chicks

**DOI:** 10.3389/fvets.2023.1152802

**Published:** 2023-03-24

**Authors:** Yunzhen Huang, Ziqiang Hu, Jiawen Dong, Linlin Li, Junqin Zhang, Ruihuan Kuang, Shimin Gao, Minhua Sun, Ming Liao

**Affiliations:** ^1^Institute of Animal Health, Guangdong Academy of Agricultural Sciences, Guangzhou, China; ^2^Key Laboratory of Livestock Disease Prevention and Treatment of Guangdong Province, Guangzhou, China; ^3^College of Veterinary Medicine Shanxi Agricultural University, Taigu, China; ^4^Guangdong Laboratory for Lingnan Modern Agriculture, Guangzhou, China; ^5^Key Laboratory for Prevention and Control of Avian Influenza and Other Major Poultry Diseases, Ministry of Agriculture and Rural Affairs, Guangzhou, China

**Keywords:** tembusu virus, egg-drop syndrome, infectivity, pathogenicity, chicks

## Abstract

In 2020, a chicken-origin Cluster 3 Tembusu virus (TMUV) caused outbreaks of a disease characterized by egg-drop syndrome in laying hens in China. In the present study, a TMUV strain, TMUV-GX, was isolated from tissue samples of laying hens with egg drop syndrome in south China. Phylogenetic analysis grouped TMUV-GX into TMUV Cluster 3.2, which was distinct from the prevalent TMUV Cluster 2 in duck flocks. To study the infectivity and pathogenicity of TMUV-GX in chickens and ducks, 7 day-old specific pathogen-free (SPF) chicks and SPF ducklings were infected with the same dose of the TMUV-GX. As a comparison, the duck-origin Cluster 2 strain, TMUV-JM, infection groups were set up in chicks and ducklings. Compared with the low infectivity and pathogenicity of TMUV-JM in chicks, the chicken-origin TMUV-GX displayed high replication competence in multiple tissues and caused tissues histopathological damage. In addition, the replication competence of TMUV-GX in ducklings was comparable to that of TMUV-JM. Our study revealed chicken-origin Cluster 3.2 TMUV exhibits high infectivity in chicks and ducklings, and suggested that chicken-origin Cluster 3.2 TMUV possesses a biological basis for widespread infection of chickens and ducks.

## Introduction

Tembusu virus (TMUV) belongs to the genus *Flavivirus* in the family *Flaviviridae* and is an enveloped virus containing positive-sense single-stranded RNA. In 1955, the first TMUV strain, MM1775, was isolated from *Culex* mosquitoes in Malaysia (1). In 2000, the first avian-origin TMUV (Sitiawan virus), causing chick encephalitis and retarded growth, was isolated in the Sitiawan District of Perak State, Malaysia (2). In subsequent years, multiple fowl-origin TMUV were identified in Southeastern Asia (3). In 2010, a TMUV-caused disease, characterized by a significant decrease in egg production of laying ducks, broke out in many duck farms in Southeastern China, and spread widely in China and Southeastern Asia in following years (4–6).

The viral full genomic length is 10990 nucleotides (nt), and contains a 5' untranslated region (UTR), a large open reading frame (ORF), and a 3' UTR. The ORF encodes a polyprotein that can be processed by viral or cellular proteases into three structural proteins [capsid (C), membrane protein (M), and envelope protein (E)] and seven non-structural proteins (NS1, NS2A, NS2B, NS3, NS4A, NS4B, and NS5). Phylogenetic analyses based on the genes encoding E and NS5 showed that the TMUVs were divided into three clusters (Cluster 1, 2, and 3). In China, TMUV isolates that circulated predominantly in water-fowl belonged to Cluster 2. Although TMUV has been reported in a variety of birds, including chickens and sparrows, the virus did not cause large-scale epidemics in these birds (7, 8).

In China, although there were a few reports of TMUV causing egg drop syndrome in laying hens (7, 9), there was no report of the widespread of TMUV in chicken flocks. In these cases, the TMUV isolates that caused egg drop syndrome in chickens belonged to TMUV Cluster 2. However, in 2020, a TMUV-caused disease associated with egg drop syndrome occurred laying hen flocks in southwest Guangdong, China (10). In a further study, researchers revealed that the TMUV isolate causing the disease belonged to TMUV Cluster 3. Subsequently, laying hen flocks infected with Cluster 3 TMUV causing egg drop syndrome were reported sequentially in Guangxi, Fujian, Hubei, Jiangsu, and Shandong, China (11). The factors that cause Cluster 3 TMUV to spread more rapidly and widely in chicken flocks than Cluster 2 TMUV remain unclear.

In this study, we isolated a Cluster 3 TMUV strain, TMUV-GX, which was identified as the pathogen of laying hen egg drop syndrome in Guangxi, China. Furthermore, We investigated the differences in infectivity and pathogenicity between TMUV-GX and the Cluster 2.2 TMUV strain TMUV-JM in both chicks and ducklings.

## Materials and methods

### Sample collection and virus isolation

In 2020, a disease characterized by a decline in egg production spread through chicken farms in many regions of south China. In this study, tissues, including livers, spleens, and ovaries, of laying hens with egg drop syndrome were collected from Guangxi, China and submitted for microbial diagnosis. For virus isolation, samples were pooled and homogenized in phosphate-buffered saline (PBS) to give a 10% suspension (wt/vol). The homogenized samples were centrifuged at 8,000 × *g* for 10 min. The supernatant was filtered through a 0.22 μm pore-size sterile filter (Millipore, Bedford, MA, USA) and inoculated into the allantoic cavity of 10-day-old specific pathogen-free (SPF) chicken embryonated eggs. Allantoic fluid of embryos that died at 2–6 days post-inoculation were collected and centrifuged. The virus was propagated three times in SPF chicken embryonated eggs.

### Viral whole genome sequencing

The viral RNA was extracted using a Tianlong Nucleic Acid Extraction & Purification Kit (T180H; Tianlong Technology Co., Ltd., Xi'an, China). First-strand cDNA was synthesized using Reverse Transcriptase ALV (Takara Biotechnology Co., Ltd., Dalian, China). To acquire the complete genome of the virus, eight pairs primers were designed based on published TMUVs sequences (Table 1). The eight PCR products were sequenced using the Sanger method by Shanghai Sangon Biotechnology Co., Ltd. (Shanghai, China). The 5'- and 3'- UTRs were amplified using a Takara SMARTer RACE 5′/3′ Kit. The complete genome was assembled using SeqMan of the DNASTAR package (DNASTAR Inc., Madison, WI, USA).

**Table 1:** Primers used in this study.

**Primer**	**Sequence**	**Size (bp)**
SEQ1F	AGAAGTTCATCTGTGTGAACTT	1,644
SEQ1R	GCTGATGACCCTGTCCAT	
SEQ2F	GTTAACAGAGAYTGGTTYCATG	1,680
SEQ2R	TTCCTTCTCATCCCACGGTC	
SEQ3F	AARRCACTRGGAGGACCAAA	1,414
SEQ3R	CCCAGATRACTCCYCCTCGT	
SEQ4F	GCGGGYTACTGGATGACAA	1,481
SEQ4R	TCCTCACTGGTWGGTCCTG	
SEQ5F	GCCAGTTCCYATAACATCAGC	1,452
SEQ5R	CTCAAGGTCYGGRACATCTGT	
SEQ6F	GGACARTTYACAATGACAACAA	1,645
SEQ6R	CATAATGCCARGAYGGGGC	
SEQ7F	CGAGATGTAYTGGGTGAGCG	1,401
SEQ7R	TGCAAYGCTGTGGCGAATCT	
SEQ8F	GTTGTGACYTAYGCTCTCAA	1,522
SEQ8R	AGACTCTGTGTTCTACCAC	
qGX-F	TGCTTTCAACATTCAGCCAGG	
qGX-R	GTTGAGCAATGTTGGCACGC	
qJM-F	AAGTGGAGCAATCAGGAAAGC	
qJM-R	AAGTGGCAGAGCAAAGGAGC	

### Phylogenetic analysis

To determine the phylogenetic relationship of the TMUV-GX strain with other reported isolates, E gene nucleotide sequences of TMUV-GX and other reference isolates were used. The nucleotide sequences were aligned, and the best nucleotide substitution model was selected using MEGA v7.0. Phylogenetic tree was generated using the maximum likelihood (ML) method implemented on MEGA v7.0 with the bootstrap method (12). The phylogenetic trees were visualized and annotated using MEGA v7.0.

### Animal experiments

To investigated the differences in infectivity and pathogenicity between TMUV-GX and TMUV-JM in both chicks and ducklings, sixty 7 day-old SPF chicks (Xinxing Dahuanong Poultry Egg Co., Ltd., Yunfu, China) and SPF ducklings (Harbin veterinary research institute, China Academy of Agricultural Sciences) were used for infection experiments. Groups of twenty chicks or twenty ducklings were intramuscularly inoculated with 10^5^ TCID_50_ (median tissue culture infectious dose) of TMUV-GX or TMUV-JM in volume of 0.1 ml. Twenty chicks and twenty ducklings were intramuscularly injected with 0.1 ml of PBS as negative controls. At 2, 4, and 6 days post-infection (dpi), three birds in each group were euthanized and liver, spleen, lung, kidney, and brain samples were collected for viral copy number detection. To investigate viral excretion, cloacal swabs and throat swabs were collected every 2 days after infection, and the mixed cloacal swabs and throat swabs samples were detected using reverse transcription quantitative real-time PCR (RT-qPCR).

For histopathological examinations, livers, kidneys, and brains were collected at 4 dpi, fixed in 4% paraformaldehyde solution, embedded in paraffin, sectioned, and stained with hematoxylin and eosin (HE).

### Total RNA isolation and RT-qPCR

Total RNA isolation from tissues and swabs was performed using RNAiso plus (Takara) according to the manufacturer's protocol. The RNA was then reverse transcribed to cDNA. To detect the copy number of TMUV RNA in samples, the cDNA was processed using the quantitative real-time PCR (qPCR) step of the RT-qPCR protocol using two pairs of qPCR primers designed for TMUV-JM and TMUV-GX NS2A genes, respectively (Table 1). Standard plasmids pMD18T-JMNS2A and pMD18T-GXNS2A, containing viral NS2A genes, were constructed and used in the assay to generate a standard curve to quantify the viral copy number. The qPCR process was carried out using a Roche LightCycler96 System (Roche Diagnostics, Basel, Switzerland) and a ChamQ SYBR qPCR Master Mix (Vazyme, Nanjing, China).

### Statistical analysis

In the figures, all data in are expressed as the mean ± standard deviation (SD). Data were analyzed using an independent-sample *t*-test in Graph Prism software (GraphPad Software Inc., San Diego, CA, USAS). Statistical significance was set at *p* < 0.05.

## Results

### Virus isolation and genome sequencing

**Figure 1:**
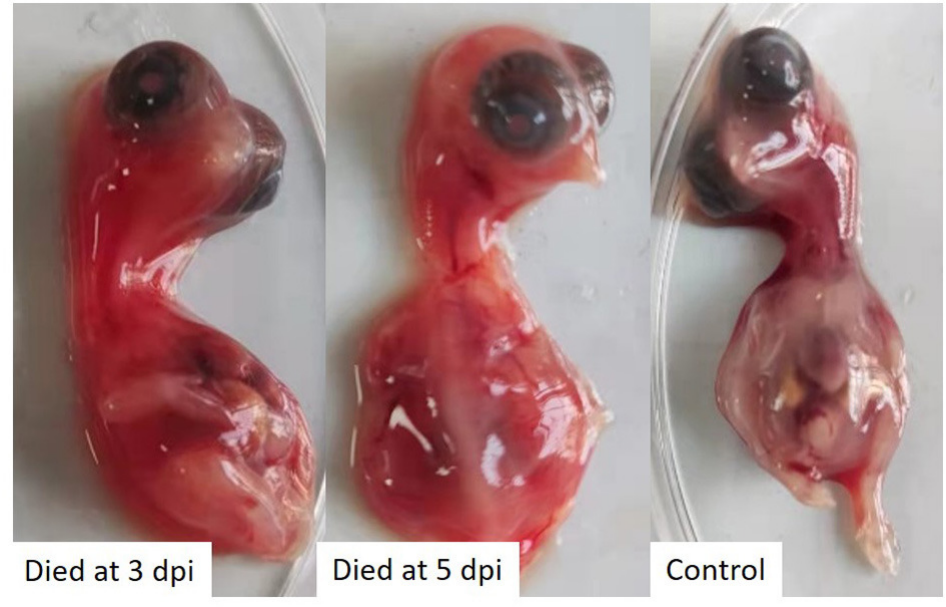
SPF chicken embryos infected with TMUV-GX died and showed severely congestion and hemorrhage.

The tissues samples collected from laying hens with egg drop syndrome were homogenized, and the supernatant tested negative for avian influenza virus, Newcastle disease virus, fowl adenovirus serotype-4, egg drop syndrome 76 virus, and infectious bursal disease virus, but tested positive for TMUV, as determined using PCR or RT-PCR assays. The virus was isolated in 10-day-old SPF chicken embryos, in which embryo death was observed. The dead embryos showed severely congestion and hemorrhage (Figure 1). The virus was propagated three times in SPF chicken embryonated eggs for purification and named as TMUV-GX.

To acquire the complete viral genome, a set of primers were used to amplify the viral genome using RT-PCR (Table 1). PCR amplicons were then sequenced. The complete genome comprised 10994 nt.

### Phylogenetic analysis

**Figure 2:**
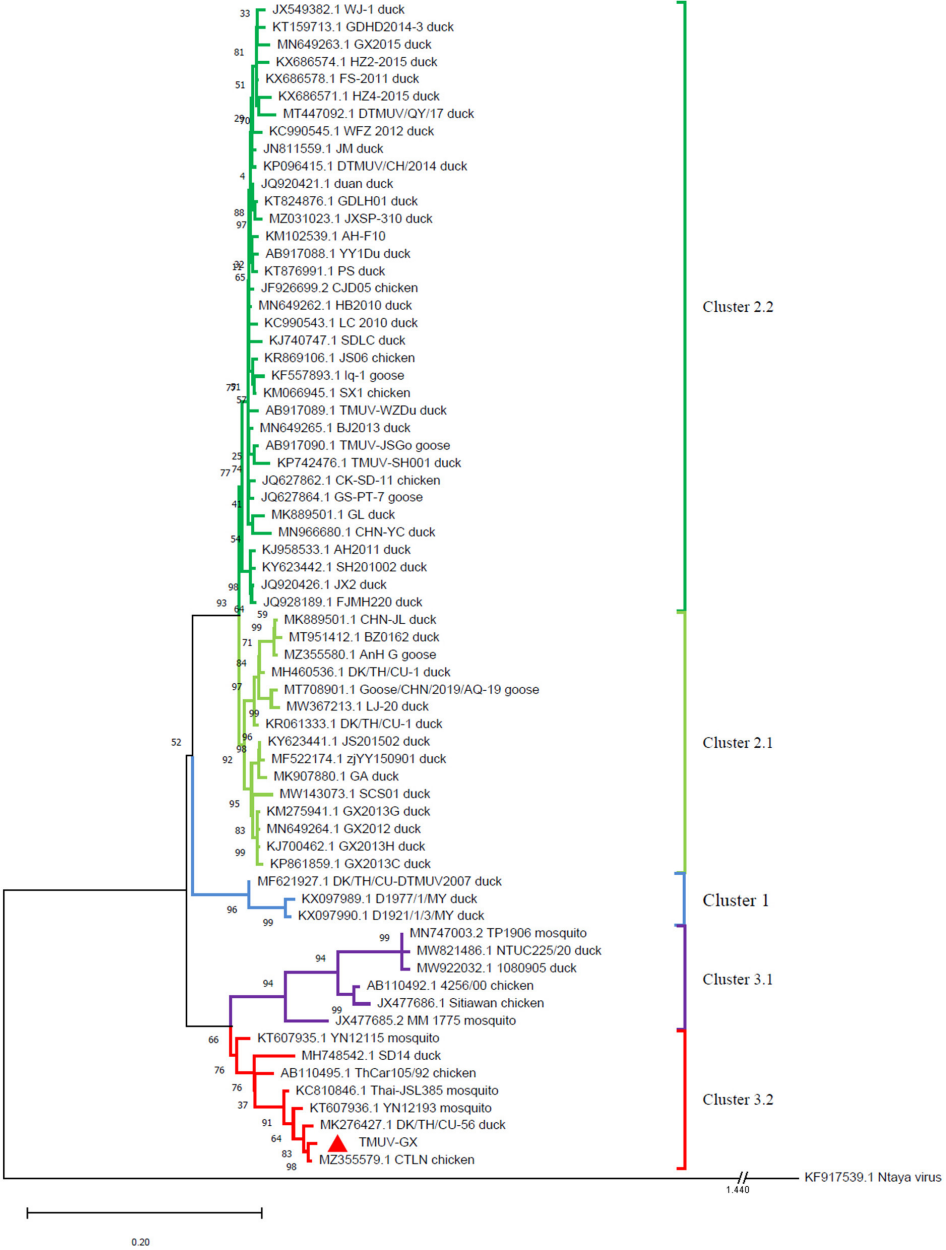
Phylogenetic analysis based on the E genes of TMUVs. The phylogenetic tree was generated *via* the ML method implement on MEGA v.7.0. Bootstrap values are shown at the nodes. The Ntaya isolate was used as the outgroup.

The evolutionary relationship between TMUV-GX and reference strains was investigated using a phylogenetic tree based on the viral E genes (Figure 2). The phylogenetic tree showed that the TMUVs were divided into three clusters. Most of the Cluster 1 TMUV strains were isolated from Southeast Asia. Cluster 2 was divided into Cluster 2.1 and Cluster 2.2, and contained most of the waterfowl-origin TMUVs in the Chinese mainland. Cluster 3 was further divided into Cluster 3.1 and Cluster 3.2. Cluster 3.1 contained mosquito-origin, duck-origin, and chicken-origin TMUVs found in Southeast Asia, but not found in the Chinese mainland. The TMUV-GX was grouped into Cluster 3.2, which contained most of the mosquito-origin TMUVs. Specifically, TMUV-GX is closely related to the TMUV CTLN strain, which causes egg-drop in chickens.

### Comparison of pathogenicity between TMUV-GX and TMUV-JM

**Figure 3:**
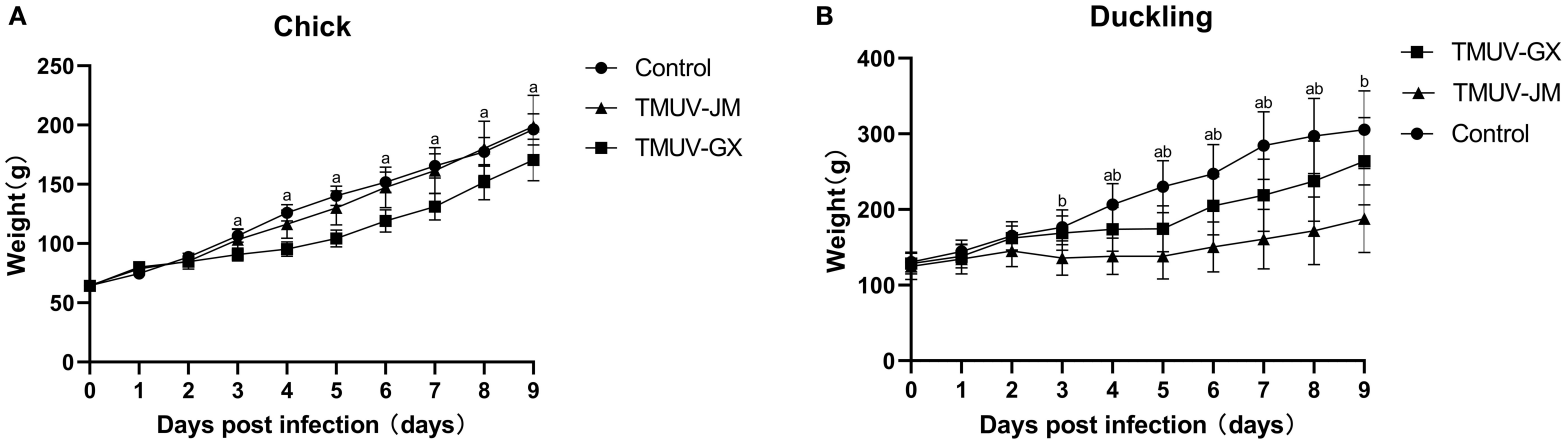
Weight changes of chicks **(A)** and ducklings **(B)** after infection with TMUV. The error bars indicate the means ± standard deviation (SD). Different lowercase letters over the bars denote statistically significant differences (*p* < 0.05) between the infection groups and the control group [(a) TMUV-GX infection group *vs*. control group; (b) TMUV-JM infection group *vs*. control group].

In the chick infection experiments, chicks in the TMUV-GX infection group did not die, but showed very mild diarrhea, moderate lack of appetite, and very mild depression (Table 2). The mean body weight in the TMUV-GX-infected chicks decreased by 20–25% compared with that in the control group at 4–7 dpi (Figure 3A). In the TMUV-JM infection group, very mild lack of appetite but no others obvious clinical signs were observed.

**Table 2:** Clinical signs in birds experimentally infected by TMUV.

**Clinical sign**	**TMUV-GX-infected chicks**	**TMUV-JM-infected chicks**	**TMUV-GX-infected ducklings**	**TMUV-JM-infected ducklings**
Diarrhea	1^a^	0	2	3
Decreased feed intake	3	1	2	4
Depression	2	0	2	3
Total	6	1	6	10

a 0 = no change; 1 = very mild; 2 = mild; 3 = moderate; 4 = marked.

In the duckling infection experiments, diarrhea, lack of appetite, and depression were observed in both the TMUV-GX and TMUV-JM infection groups (Table 2). No ducks died during the experiment. However, the clinical signs were more severe in the TMUV-JM infection group than in the TMUV-GX infection group. Compared with that in the control group, the body weights gain in TMUV-infected ducklings decreased significantly after infection. Specifically, compared with that in the control group, the mean body weight of the TMUV-GX-infected ducklings decreased by 15–25% at 4–8 dpi, and the mean body weight of the TMUV-JM-infected ducklings decreased by 33–45% at 4–9 dpi (Figure 3B).

**Figure 4:**
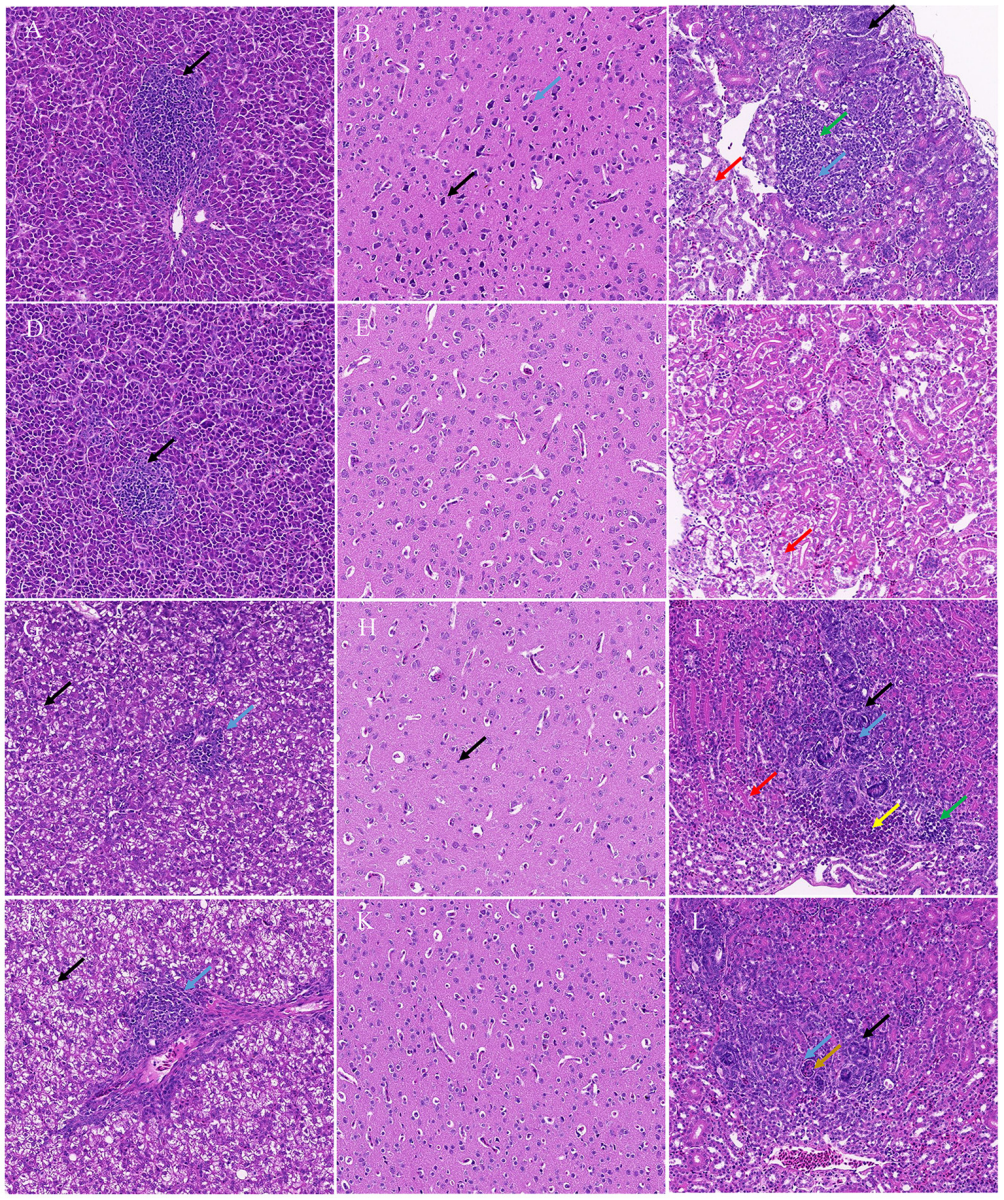
Histopathological lesions. **(A)** Liver, TMUV-GX-infected chicks: lymphoid infiltration (black arrow); **(B)** Brain, TMUV-GX-infected chicks: neuronal pyknosis, enhanced basophilic cytoplasm (black arrow) and increase in microglia (blue arrow); **(C)** Kidney, TMUV-GX-infected chicks: renal tubular atrophy, luminal stenosis (black arrow), multiple epithelial cell necrosis, nuclear pyknosis (blue arrow), lymphoid infiltration (green arrow), and hydropic degeneration of renal tubular epithelial cells (red arrow); **(D)** Liver, TMUV-JM-infected chicks: lymphoid infiltration (black arrow); **(E)** Brain, TMUV-JM-infected chicks; **(F)** Kidney, TMUV-JM-infected chicks: some hydropic degeneration of renal tubular epithelial cells (red arrow); **(G, J)** Liver, TMUV-infected ducklings: diffuse hydropic degeneration of hepatocytes (black arrow) and some lymphoid infiltration (blue arrow); **(H)** Brain, TMUV-GX-infected ducklings: increase in microglia (black arrow); **(I)** Kidney, TMUV-GX-infected ducklings: renal tubular atrophy, luminal stenosis (black arrow), multiple epithelial cell necrosis, nuclear pyknosis (blue arrow), lymphoid infiltration (green arrow), hydropic degeneration of renal tubular epithelial cells (red arrow), and hemorrhage (yellow arrow); **(K)** Brain, TMUV-JM-infected ducklings; **(L)** Kidney, TMUV-JM-infected ducklings: renal tubular atrophy, luminal stenosis (black arrow), multiple epithelial cell necrosis, nuclear pyknosis (blue arrow), and necrotic cell mass (brown arrow).

Histopathology revealed that the chicks and ducklings inoculated with TMUV-GX presented significant lesions in the liver, brain, and kidney. In the liver, multifocal lymphoid infiltration was frequently observed in the TMUV-GX-infected chicks, while lymphoid infiltration was less frequently observed in the TMUV-JM-infected chicks (Figures 4A, D). In TMUV-infected ducklings, there were lesions in the liver characterized by diffuse hydropic degeneration of hepatocytes and some lymphoid infiltration (Figures 4G, J). In the brain, we observed neuronal pyknosis, enhanced basophilic cytoplasm, and increase of microglia in the TMUV-GX-infected chicks. Increased microglia were also observed in the TMUV-GX-infected ducklings, whereas no obvious lesions were found in the TMUV-JM-infected chicks and ducklings (Figures 4B, E, H, K). In the kidney, renal tubular atrophy, luminal stenosis, multiple epithelial cell necrosis, nuclear pyknosis, lymphoid infiltration, and hydropic degeneration of renal tubular epithelial cells were observed in the TMUV-GX-infected chicks and ducklings (Figures 4C, I). There were lesions characterized by renal tubular atrophy, luminal stenosis, epithelial cell necrosis, nuclear pyknosis in the kidneys of the TMUV-JM-infected ducklings, whereas less hydropic degeneration of renal tubular epithelial cells was observed in the TMUV JM infected chicks (Figures 4F, L).

### Viral RNA copy number in tissues and viral excretion

**Figure 5:**
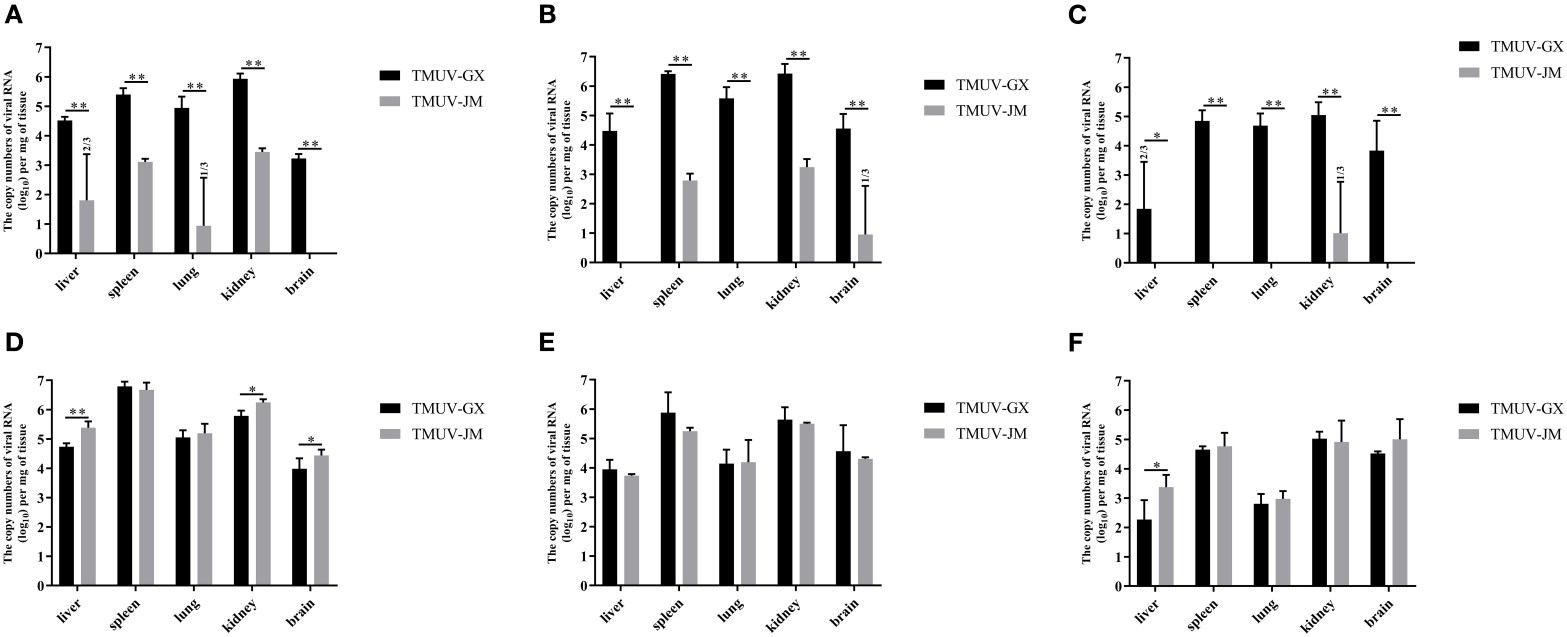
Viral RNA copy numbers in different tissues of experimentally infected animals. **(A–C)** show the viral RNA copy numbers in tissue samples of the TMUV-infected chicks at 2, 4, and 6 dpi, respectively. **(D–F)** show the viral RNA copy numbers in tissue samples of the TMUV-infected ducklings at 2, 4, and 6 dpi, respectively. The statistical differences between both groups were evaluated using an independent-sample *t*-test. *p < 0.05, **p < 0.01.

The virus loads in the different tissues of TMUV-infected chicks and ducklings were determined using RT-qPCR (Figure 5). In the chick infection experiments (Figures 5A–C), the viral RNA could be detected in all investigated tissues of the TMUV-GX infection group at 2 and 4 dpi, whereas only the spleens and kidneys of the TMUV-JM infection group were positive for viral RNA at 2 and 4 dpi. Viral RNA copy numbers in all investigated tissues obtained from the TMUV-GX infection group were significantly higher than those in the TMUV-JM infection group. The maximum viral RNA copy numbers were achieved at 4 dpi, and higher viral RNA copy numbers were found in the kidneys and spleen than in other tissues. Furthermore, viral RNA copy numbers in the brains of the TMUV-GX infection group were generally higher at 4 and 6 dpi, whereas viral RNA was not detected in the brain of most TMUV-JM-infected chicks. In the duckling infection experiments (Figures 5D-5F), the viral RNA could be detected in all investigated tissues of the TMUV-infected ducklings at 2, 4, and 6 dpi, with the maximum viral RNA copy numbers being achieved at 2 dpi. Furthermore, viral RNA copy numbers in the liver, kidney and brain were lower in the TMUV-GX infection group than in the TMUV-JM infection group at 2 dpi. No viral RNA was detected in the control groups. Generally, the viral copy numbers in the investigated tissues from the TMUV-GX-infected chicks were higher than those in the TMUV-JM-infected chicks, but did not differ between the two duckling infected groups.

To investigate viral excretion, cloacal swabs and throat swabs were collected at 2, 4, and 6 dpi, and the mixed cloacal swabs and throat swab samples were detected using RT-qPCR (Table 3). Swabs collected from TMUV-GX-infected chicks and ducklings were detected as 100% positive for viral RNA at 2, 4, and 6 dpi. Viral RNA was also detected in swabs collected from TMUV-JM-infected ducklings at 2, 4, and 6 dpi. However, swabs collected from TMUV-JM-infected chicks were negative for viral RNA at 2, 4, and 6 dpi. No positive viral RNA was recorded in the control birds in this study.

**Table 3:** Detection of virus in swab samples using RT-qPCR.

**Day post-infection**	**Chicks**		**Ducklings**	
	**TMUV-GX**	**TMUV-JM**	**TMUV-GX**	**TMUV-JM**
2	10/10^a^	0/10	10/10	10/10
4	10/10	0/10	10/10	10/10
6	10/10	0/10	10/10	10/10

a Number of positive swabs/number of birds from infected group.

## Discussion

Generally, TMUV has been considered as highly contagious in water-fowl (13). Although TMUV-induced egg-drop sporadic cases in laying hens have been reported, there has been no report widespread of TMUV infections in chicken flocks. Since 2020, Cluster 3 TMUV infection causing laying hen egg drop syndrome occurred successively in laying hen flocks in many provinces of China. In this study, a Cluster 3 TMUV isolate, TMUV-GX, was isolated from samples of laying hens with egg drop syndrome.

The E gene plays an important role in viral antigenicity, tissue tropism, transmissibility, and virulence (14–16). The result of phylogenetic analysis based on the E gene showed that the TMUVs were divided into 3 clusters. Cluster 1 and 2 mainly contained water-fowl origin isolates. Although Cluster 2 TMUVs were sporadically isolated from other birds (including chickens, sparrows, and pigeons), they did not cause an epidemic in these birds. The TMUV-GX, isolated in this study, was phylogenetically grouped into Cluster 3.2, which contained most of the mosquito-origin TMUVs. Furthermore, TMUV-GX was most closely related to the CTLN strain, which was isolated from laying hen flocks displaying clinical egg drop syndrome in Guangdong, China, in 2020. We believe that TMUV-GX and CTLN are different isolates that caused the same epidemic that occurred successively in many regions of China. In addition, TMUV-GX was also clustered with the mosquito-origin YN12193 strain and the duck-origin DK/TH/CU-56 strain. Therefore, we conjectured that the Cluster 3.2 TMUV, which spread widely in chicken flocks recently, could become a potential threat for both fowl and water-fowl flocks.

We compared the infectivity and pathogenicity of TMUV-GX with that of duck-origin Cluster 2.2 strain TMUV-JM in chicks and ducks. TMUV-GX-infected chicks displayed clinical signs, consisting of diarrhea, lack of appetite, depression, and growth retardation, which were not observed in the TMUV-JM-infected chicks. The same clinical signs were observed in TMUV-GX-infected and TMUV-JM-infected ducklings. The histological lesions observed in the TMUV-GX-infected ducklings were similar to those of TMUV-JM-infected ducklings, except for the brain. However, the lesions in chicks attributed to TMUV-GX infection were more serious and more numerous, especially in the brain, compared with those in TMUV-JM infection. The clinical signs and microscopic lesions indicated that the TMUV-GX is more pathogenic for chicks than TMUV-JM. The results of tissue viral RNA load determination showed significantly higher viral copy numbers in all investigated tissues of TMUV-GX-infected chicks in comparison with TMUV-JM-infected chicks, demonstrating that the replication competence of TMUV-GX in chicks was stronger than that of TMUV-JM. However, the levels of tissue viral RNA load of TMUV-GX-infected and TMUV-JM-infected ducklings were almost identical, indicating that the replication competence of TMUV-GX in ducklings is comparable to that of TMUV-JM.

## Conclusion

The results of the present study demonstrated that the chicken-origin TMUV-GX has high infectivity and certain pathogenicity toward chicks, whereas duck-origin cluster 2 TMUV did not have these characteristics. In addition, we revealed that chicken-origin Cluster 3.2 TMUV has high infectivity and certain pathogenicity toward ducklings. Our findings suggested that Cluster 3.2 TMUV, which has spread widely in chicken flocks recently, could be a potential threat to both chicken and duck flocks.

## Data availability statement

The original contributions presented in the study are included in the article/Supplementary material, further inquiries can be directed to the corresponding authors.

## Ethics statement

The animal study was reviewed and approved by the Committee on the Ethics of Animal Experiments of Institute of Animal Health, Guangdong Academy of Agricultural Sciences Experimental Animal Welfare Ethics Committee (Approve ID: 2018-007).

## Author contributions

SG, MS, and ML supervised this project, designed the experiments. YH, ZH, JD, LL, JZ, and RK collected samples and performed experiments. YH analyzed data and prepared manuscript. All authors read and approved the final manuscript.
