# Supplementary figures and images for: Chicken-origin Cluster 3.2 Tembusu virus exhibits higher infectivity than duck-origin Cluster 2 Tembusu virus in chicks
Source: Front Vet Sci. 2023 Mar 24;10:1152802. doi: 10.3389/fvets.2023.1152802 (PMC10080150; doi:10.3389/fvets.2023.1152802)

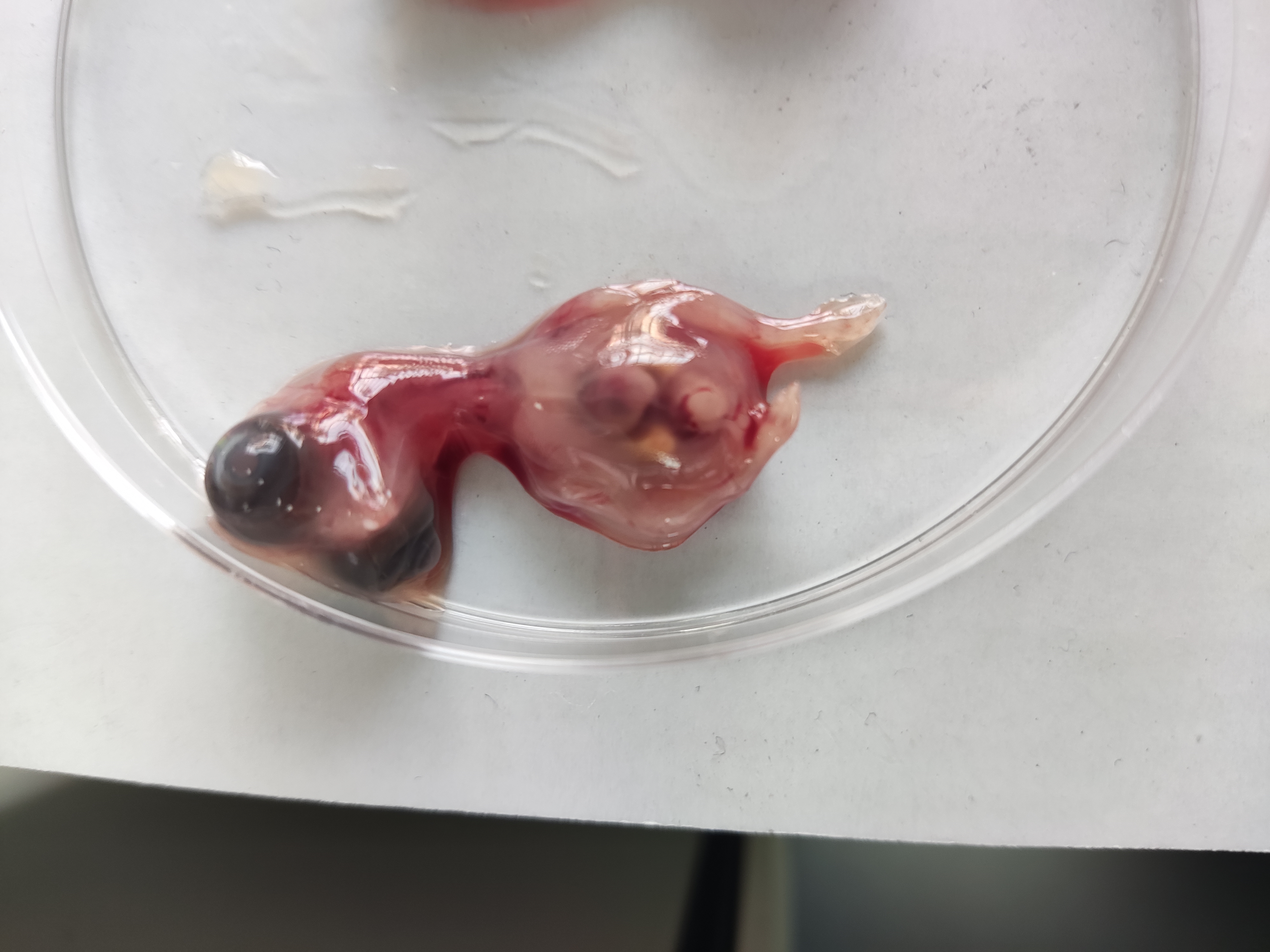

Supplement: Supplementary file 1 [file Data_Sheet_1.ZIP › chicken embryos infected with TMUV-GX/control.jpg]

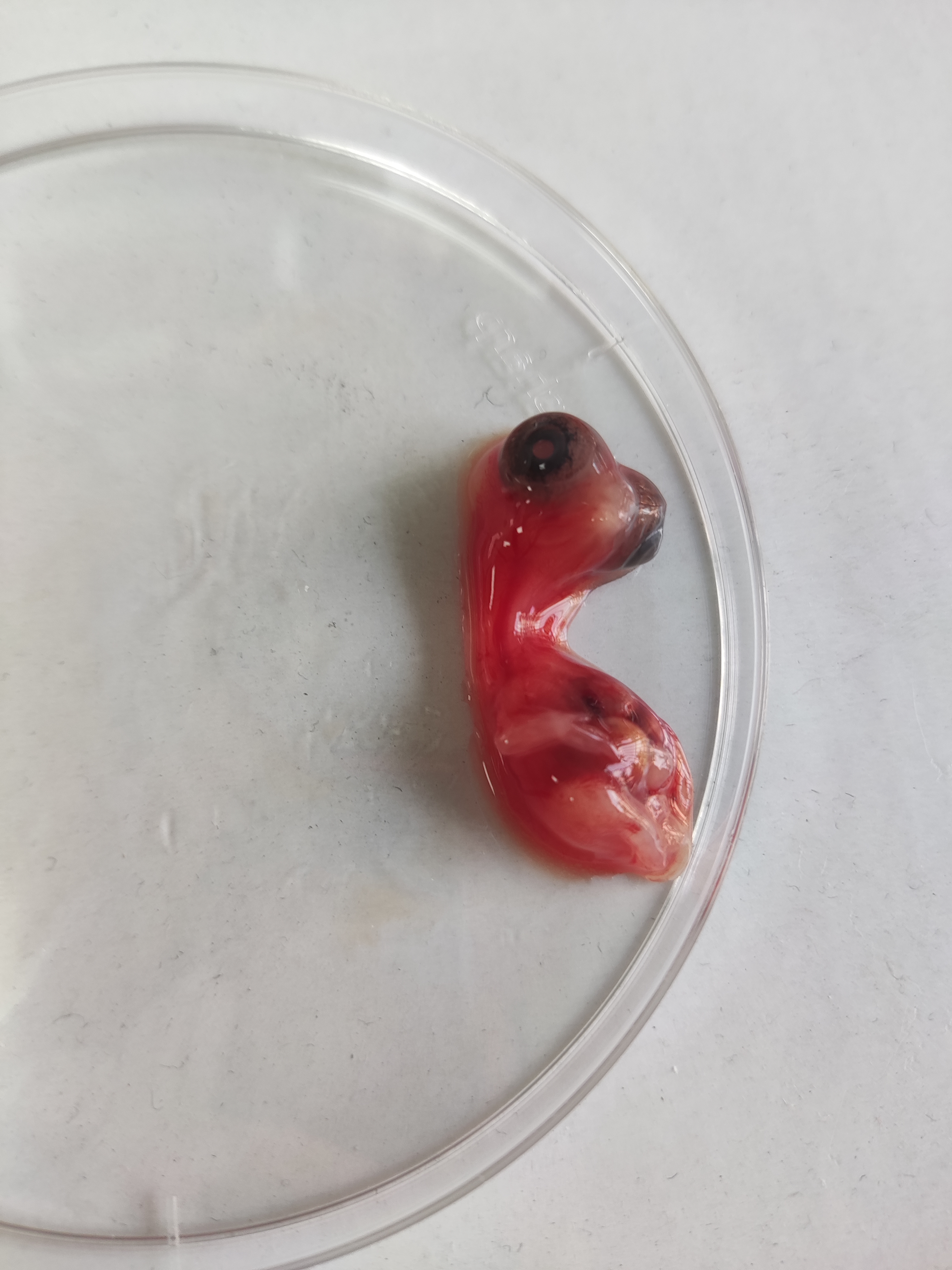

Supplement: Supplementary file 1 [file Data_Sheet_1.ZIP › chicken embryos infected with TMUV-GX/died at 3 dpi.jpg]

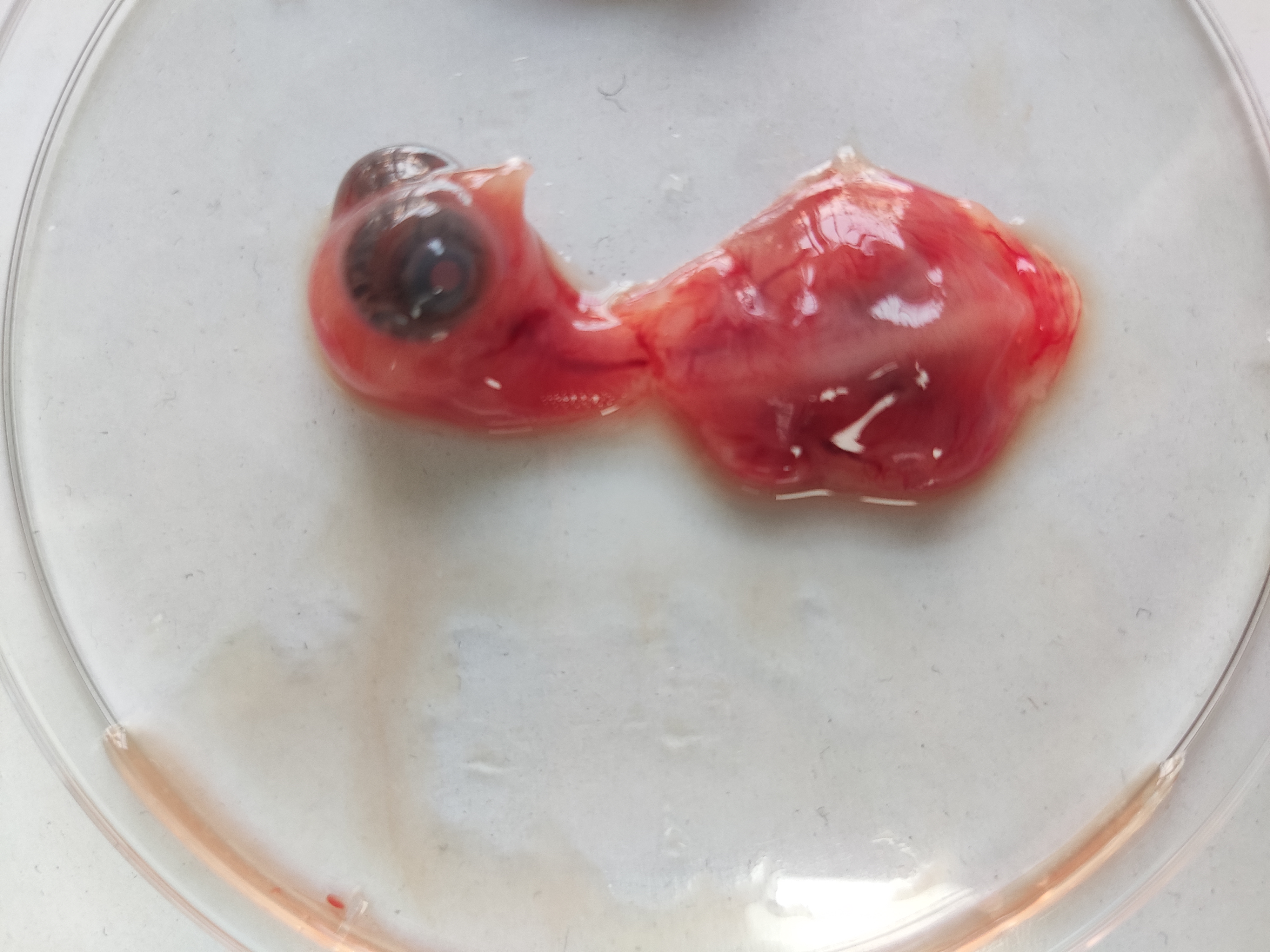

Supplement: Supplementary file 1 [file Data_Sheet_1.ZIP › chicken embryos infected with TMUV-GX/died at 5 dpi.jpg]

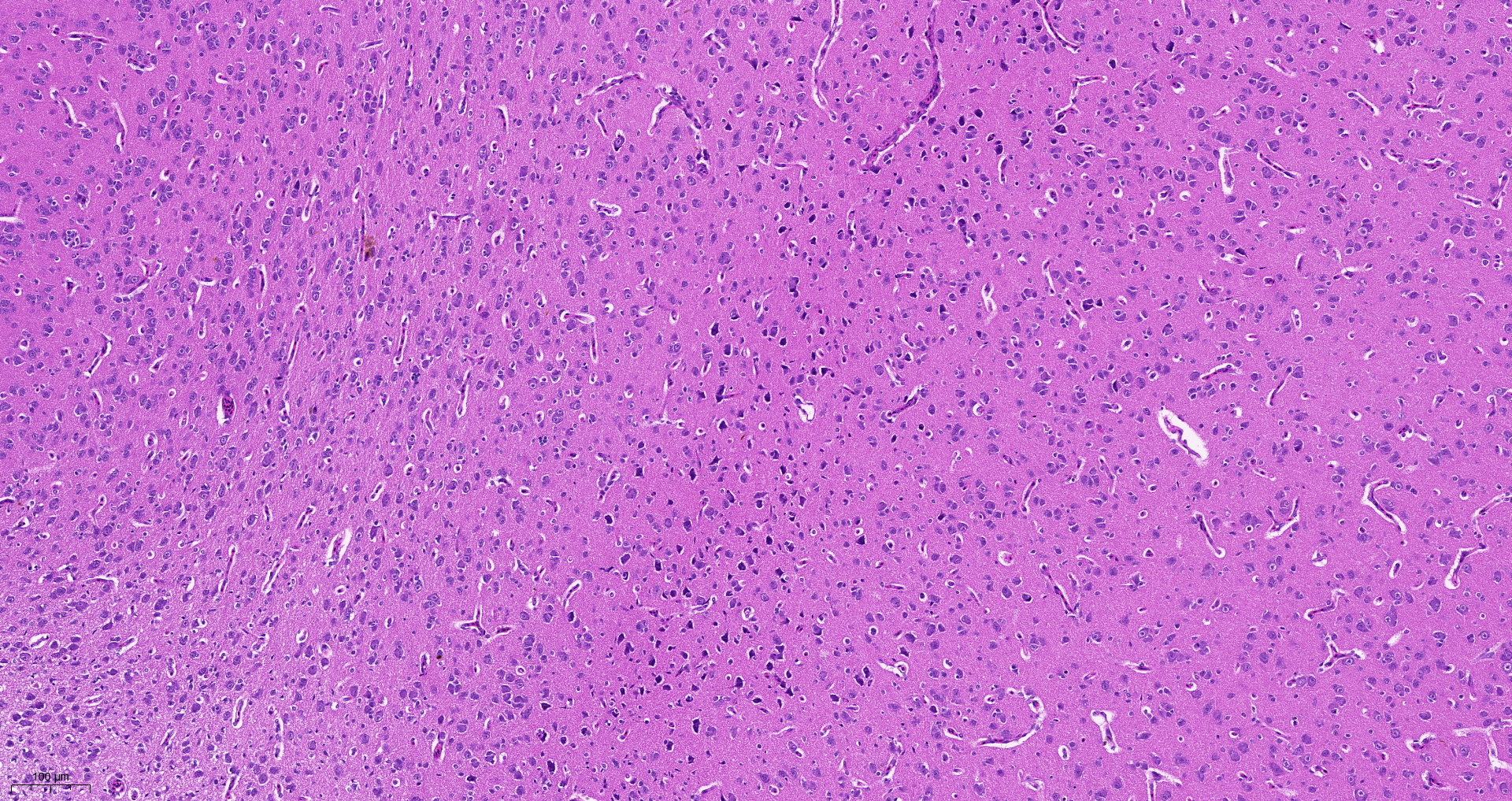

Supplement: Supplementary file 2 [file Data_Sheet_2.ZIP › chicken 4dpi TMUV GX Brain_10.0x.jpg]

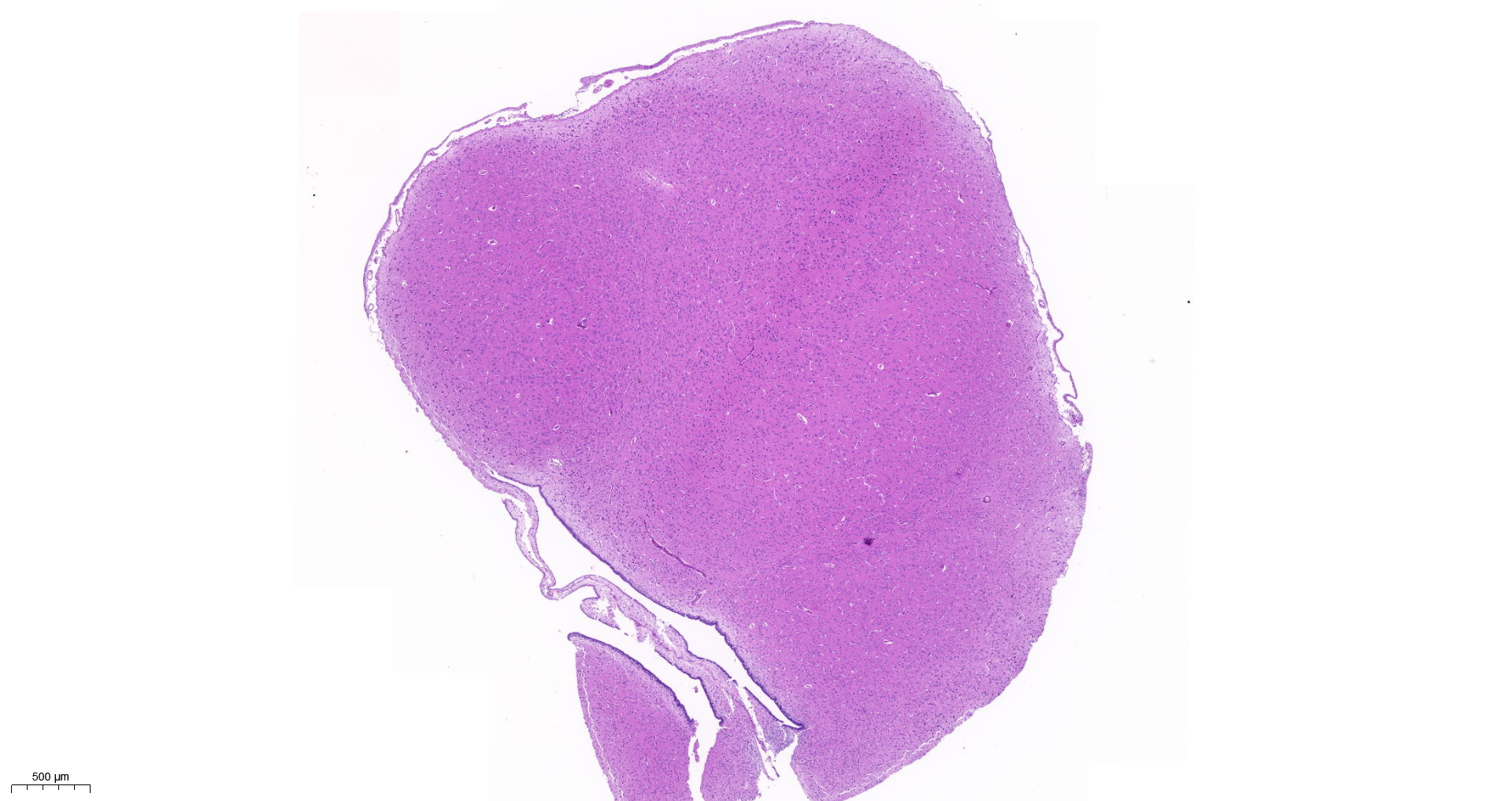

Supplement: Supplementary file 2 [file Data_Sheet_2.ZIP › chicken 4dpi TMUV GX Brain_2.0x.jpg]

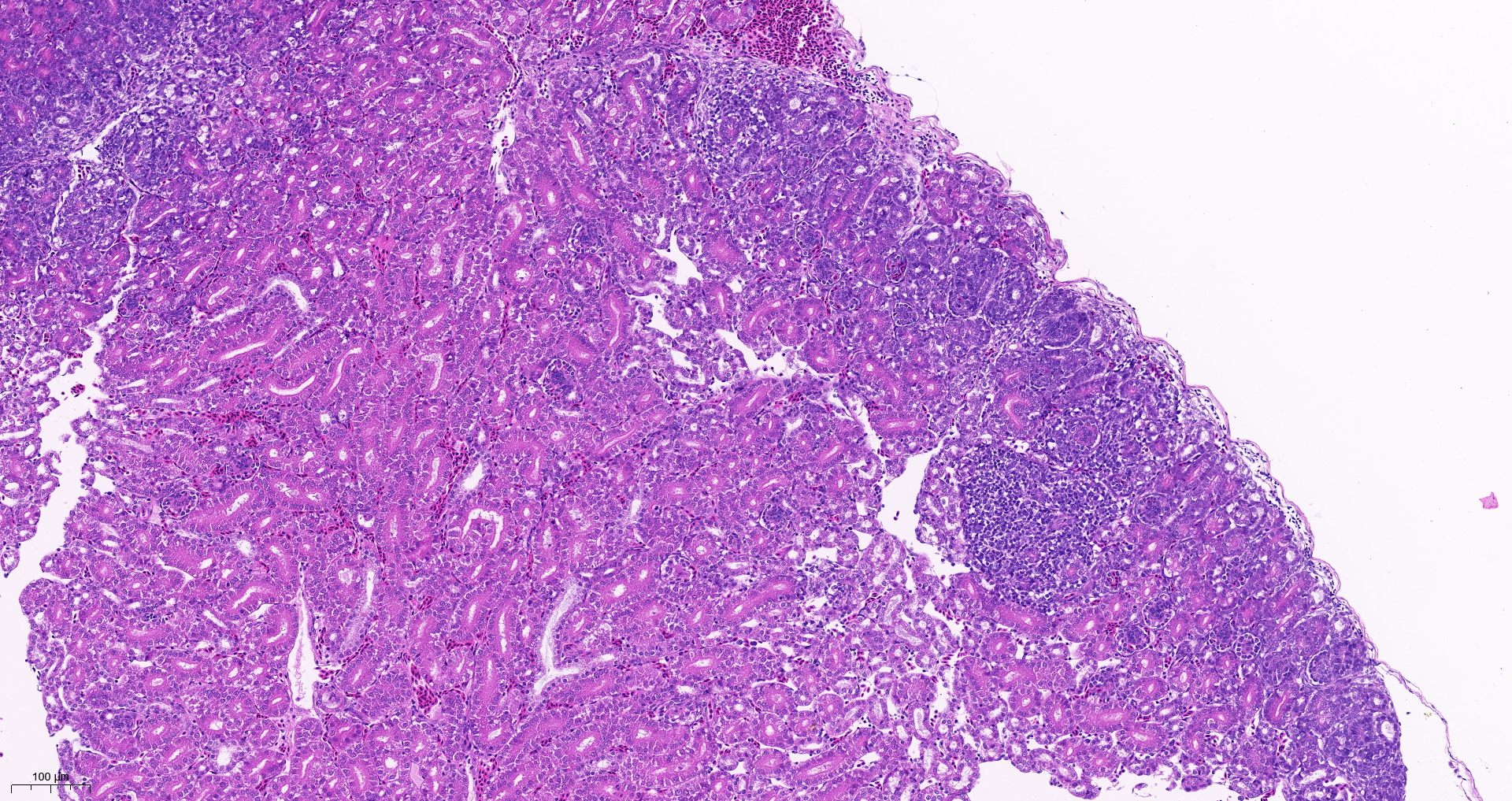

Supplement: Supplementary file 2 [file Data_Sheet_2.ZIP › chicken 4dpi TMUV GX Kidney_10.0x.jpg]

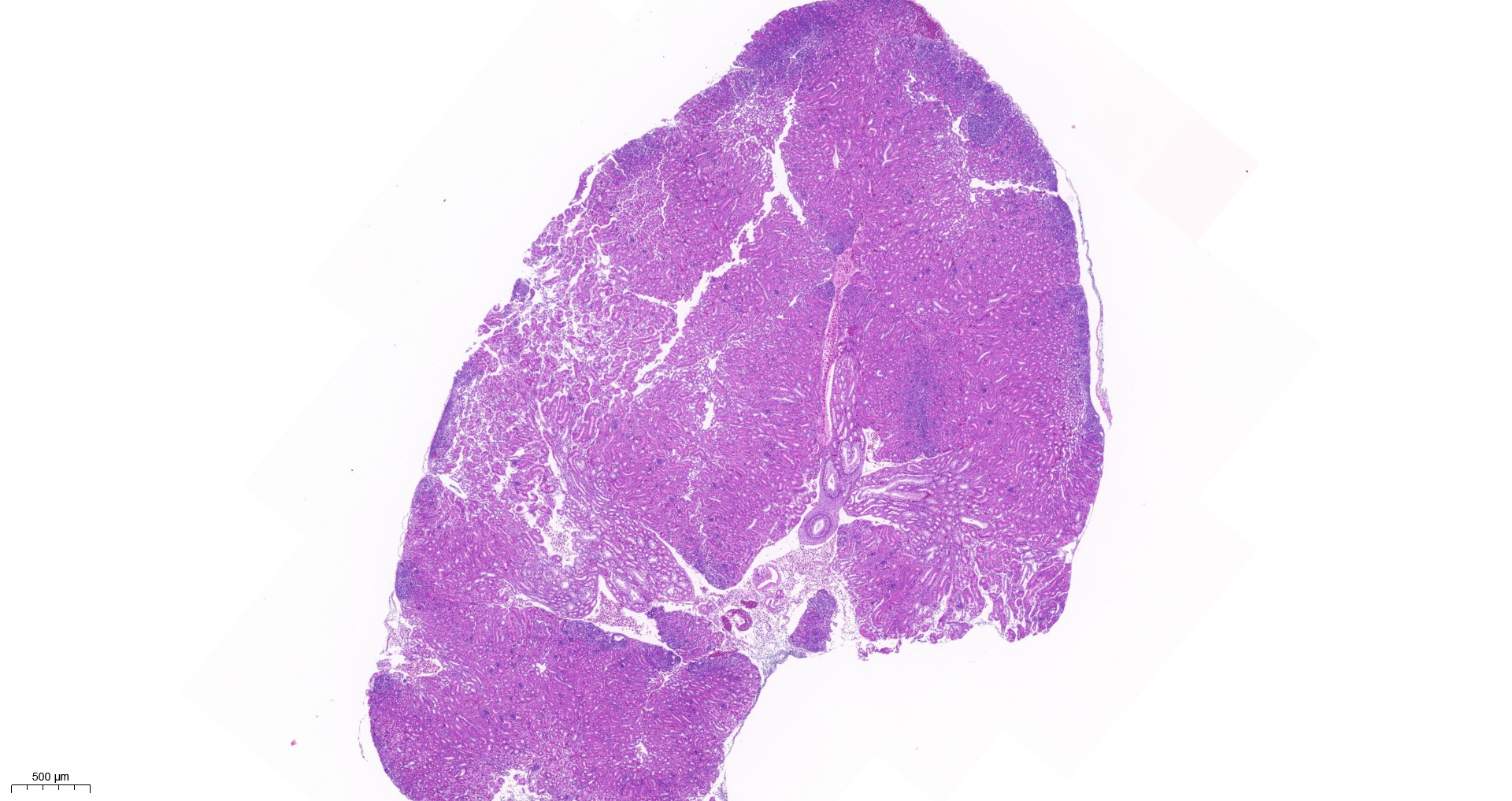

Supplement: Supplementary file 2 [file Data_Sheet_2.ZIP › chicken 4dpi TMUV GX Kidney_2.0x.jpg]

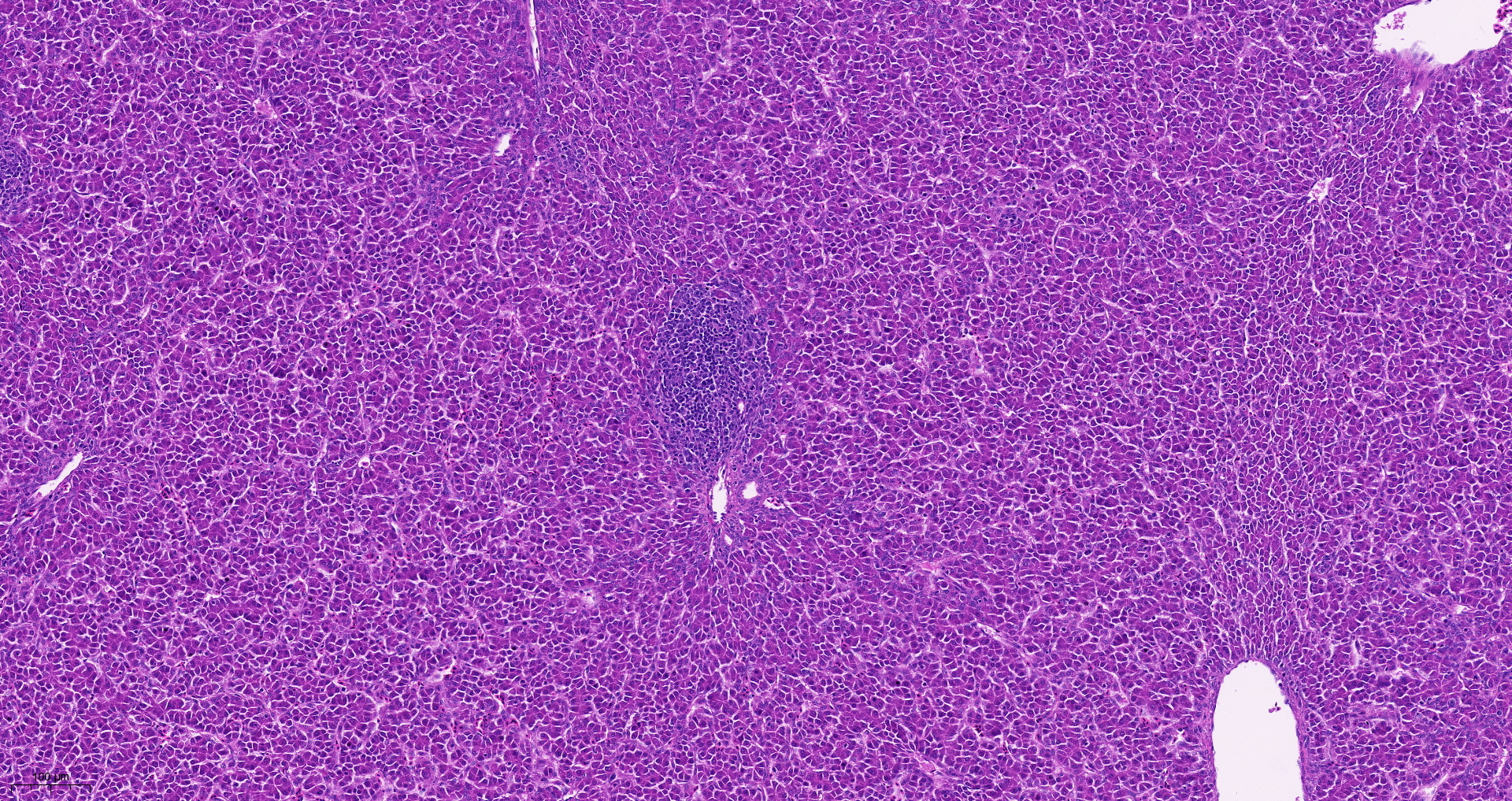

Supplement: Supplementary file 2 [file Data_Sheet_2.ZIP › chicken 4dpi TMUV GX liver_10.0x.jpg]

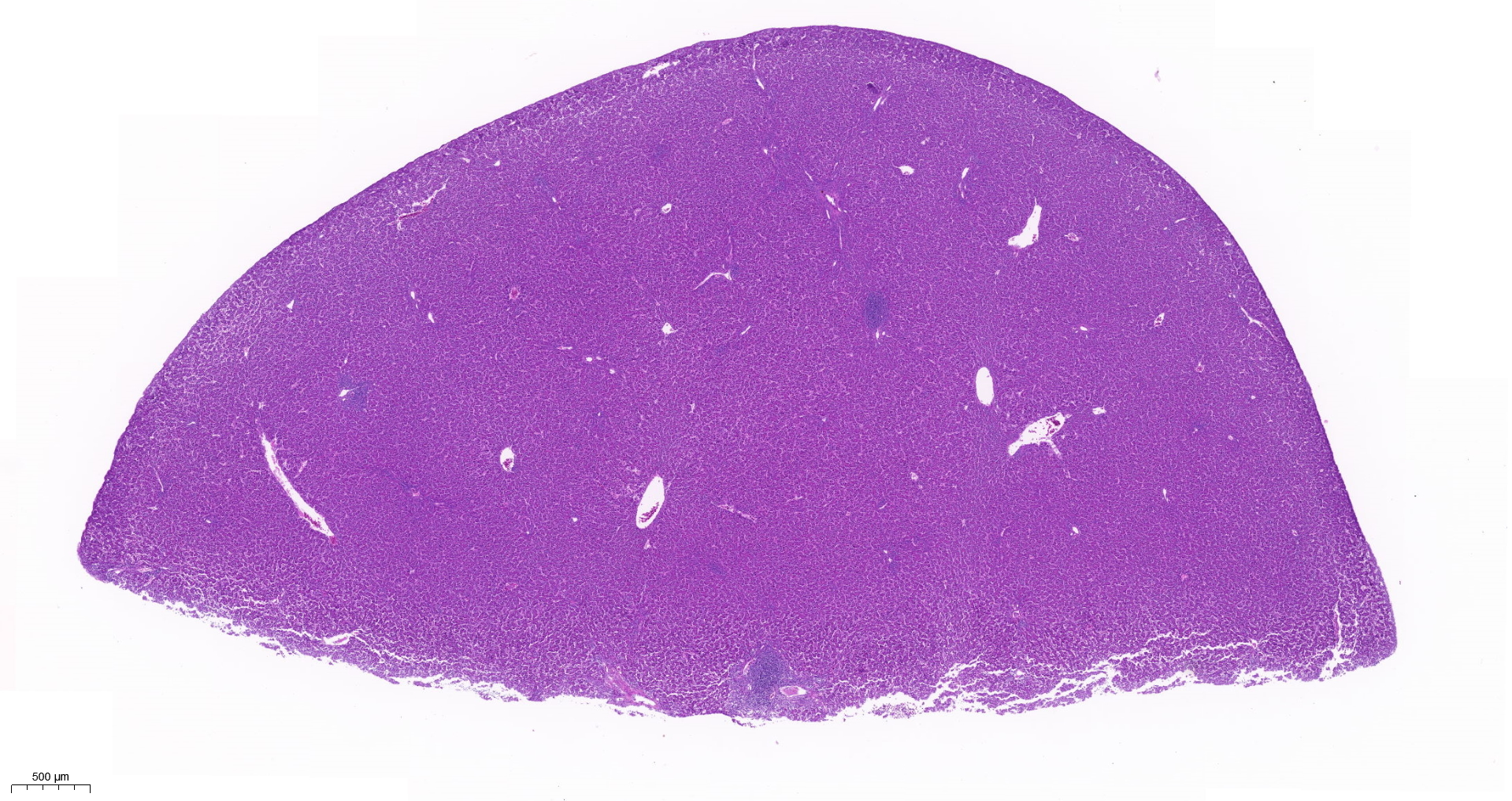

Supplement: Supplementary file 2 [file Data_Sheet_2.ZIP › chicken 4dpi TMUV GX liver_2.0x.jpg]

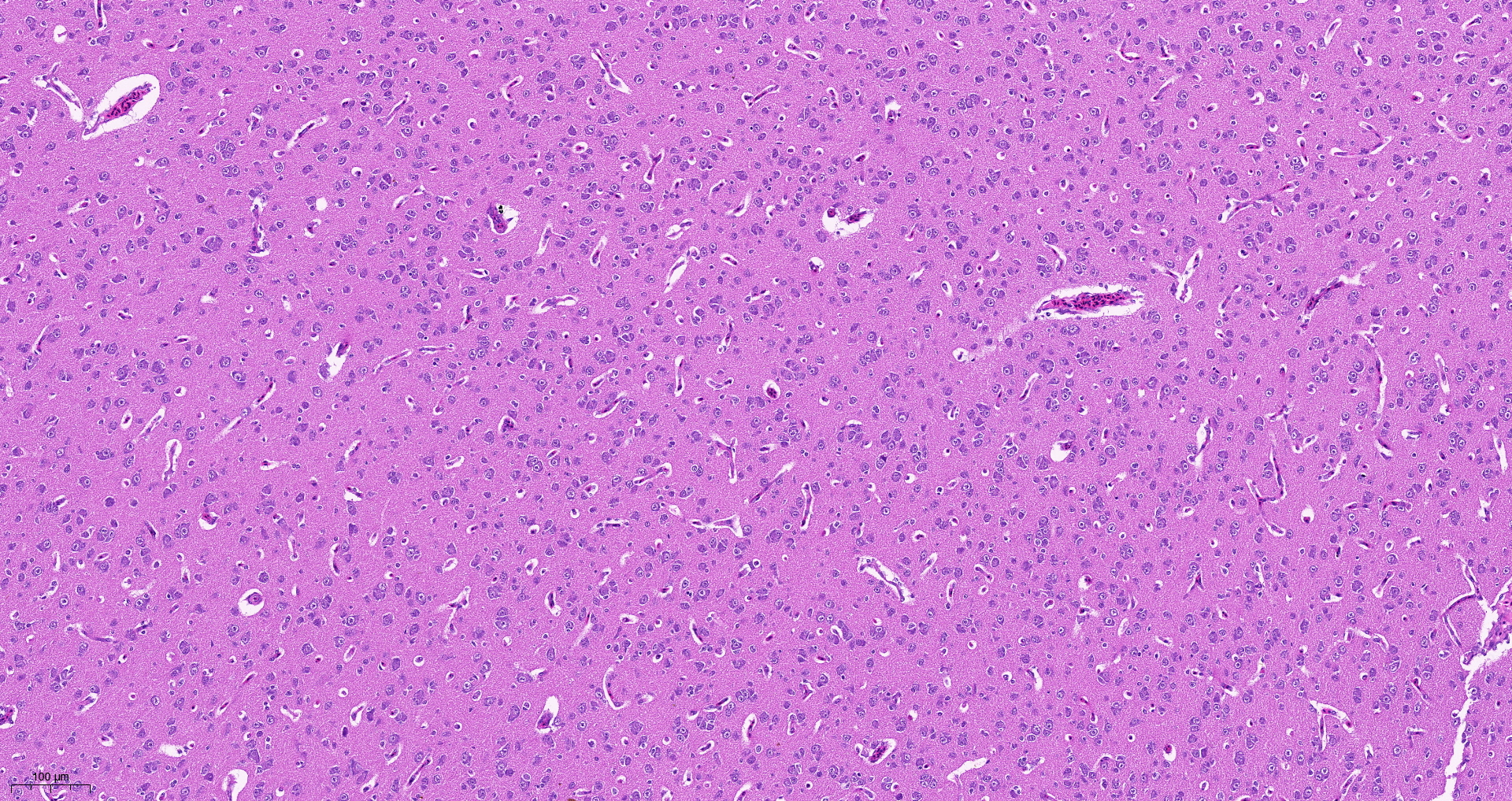

Supplement: Supplementary file 2 [file Data_Sheet_2.ZIP › chicken 4dpi TMUV JM Brain_10.0x.jpg]

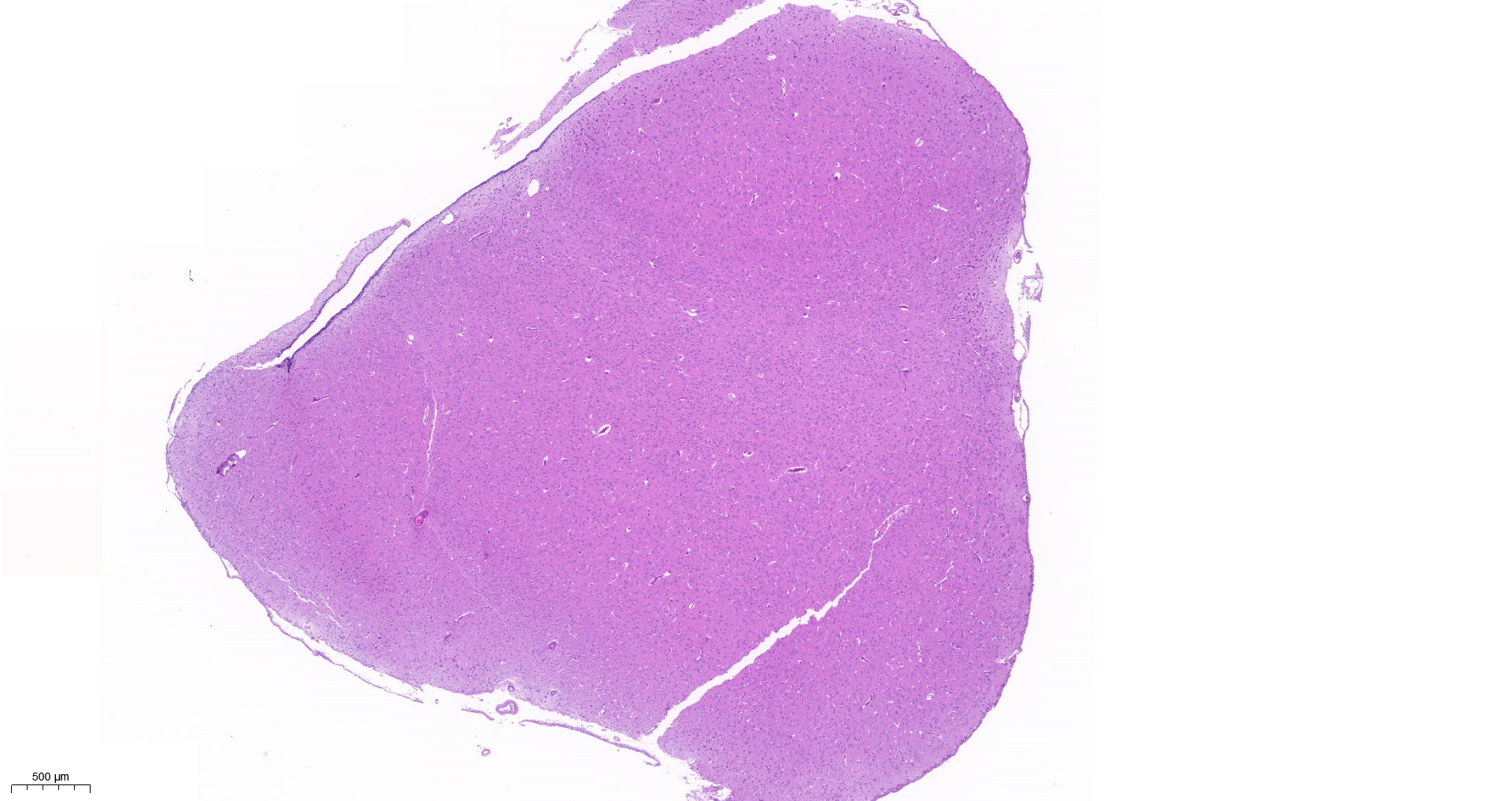

Supplement: Supplementary file 2 [file Data_Sheet_2.ZIP › chicken 4dpi TMUV JM Brain_2.0x.jpg]

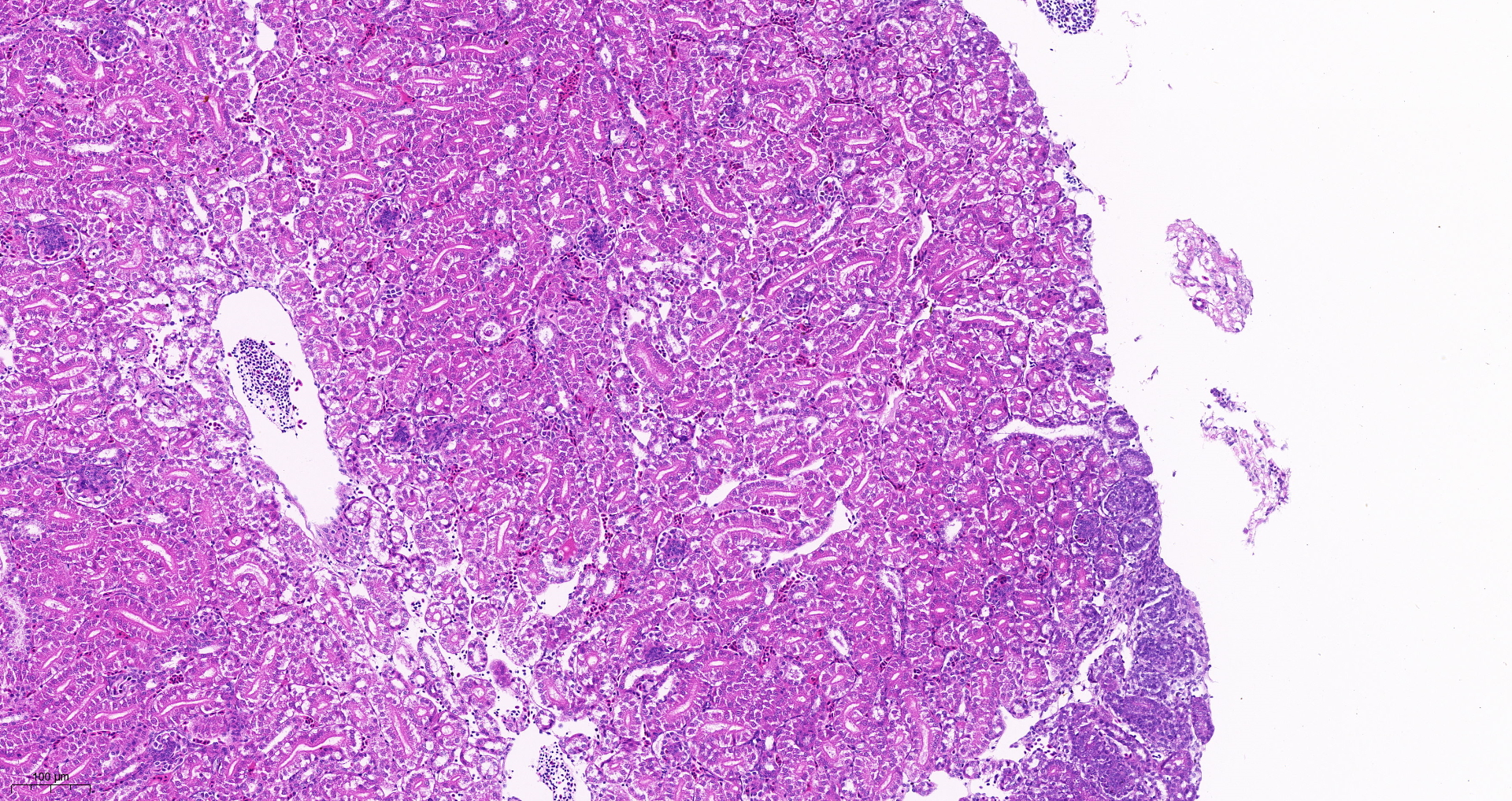

Supplement: Supplementary file 2 [file Data_Sheet_2.ZIP › chicken 4dpi TMUV JM Kidney_10.0x.jpg]

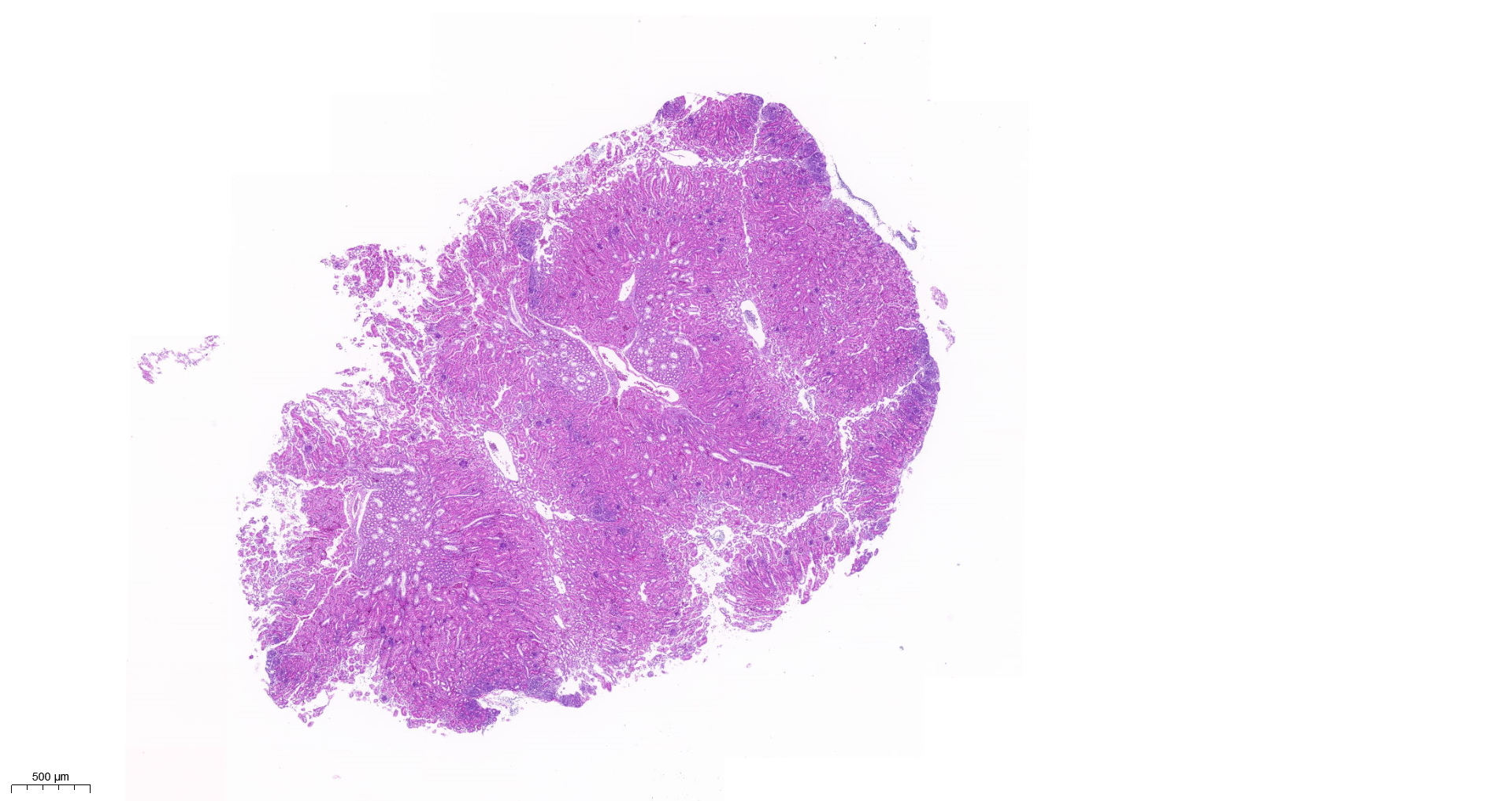

Supplement: Supplementary file 2 [file Data_Sheet_2.ZIP › chicken 4dpi TMUV JM Kidney_2.0x.jpg]

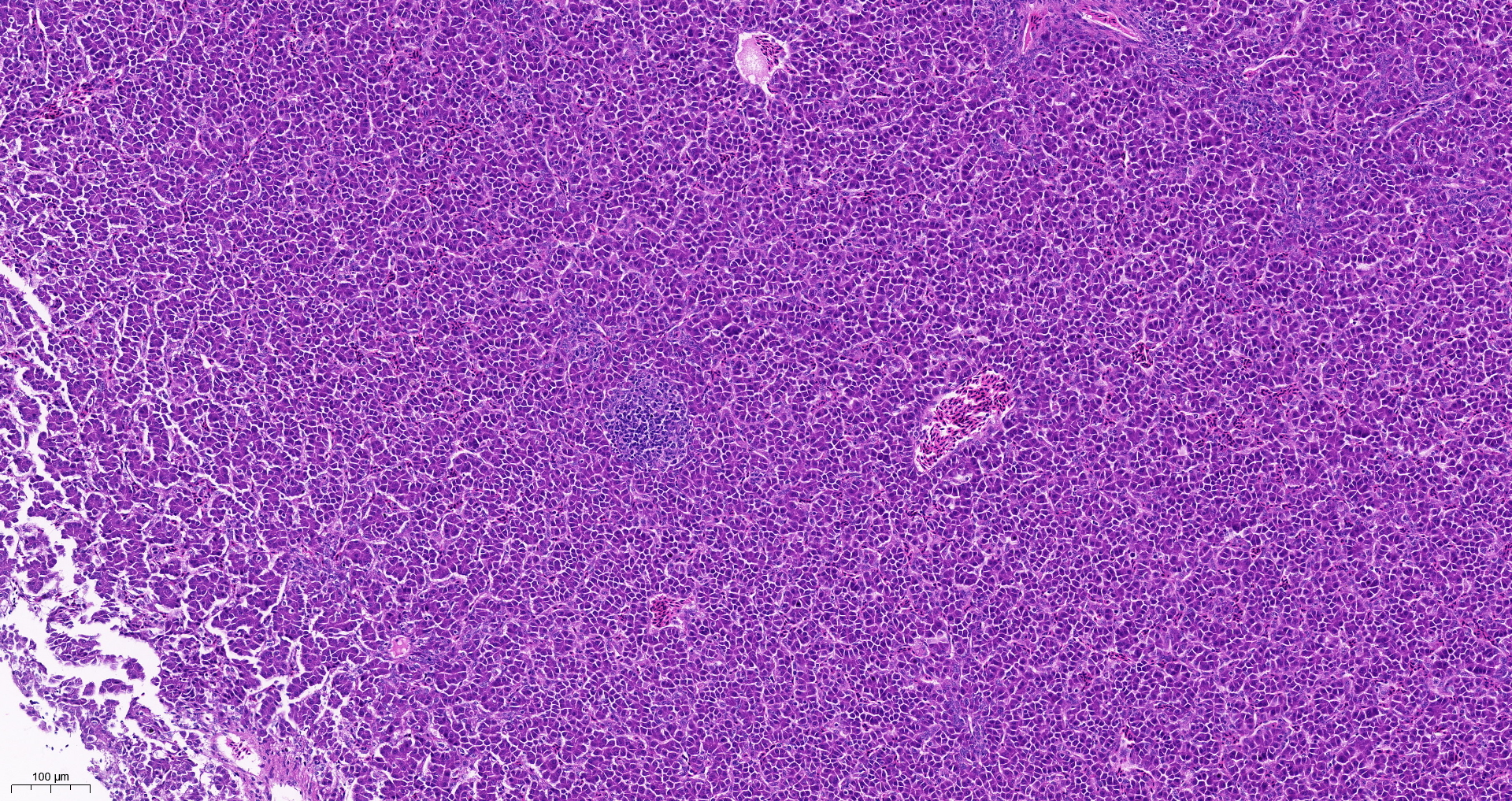

Supplement: Supplementary file 2 [file Data_Sheet_2.ZIP › chicken 4dpi TMUV JM liver_10.0x.jpg]

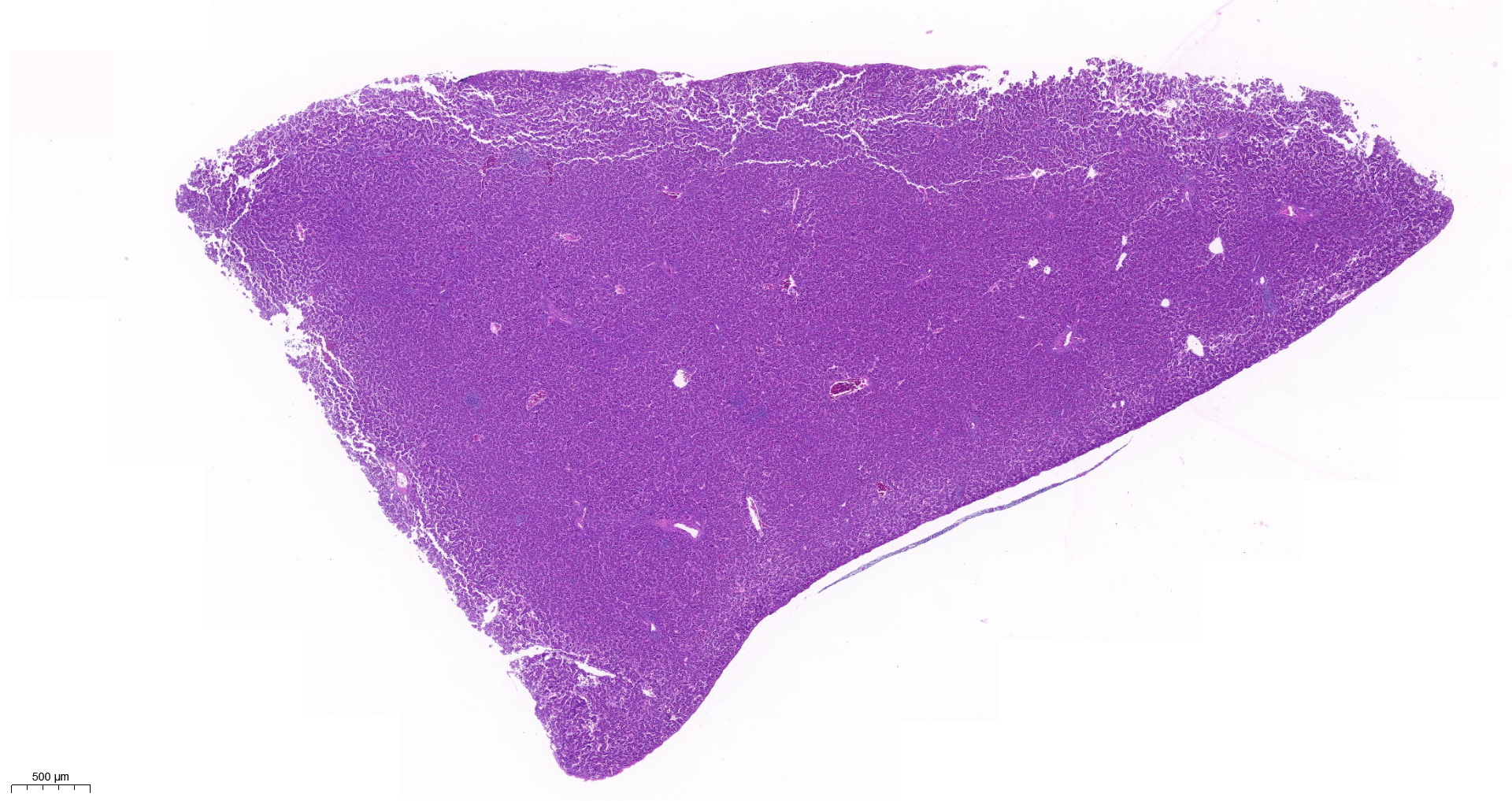

Supplement: Supplementary file 2 [file Data_Sheet_2.ZIP › chicken 4dpi TMUV JM liver_2.0x.jpg]

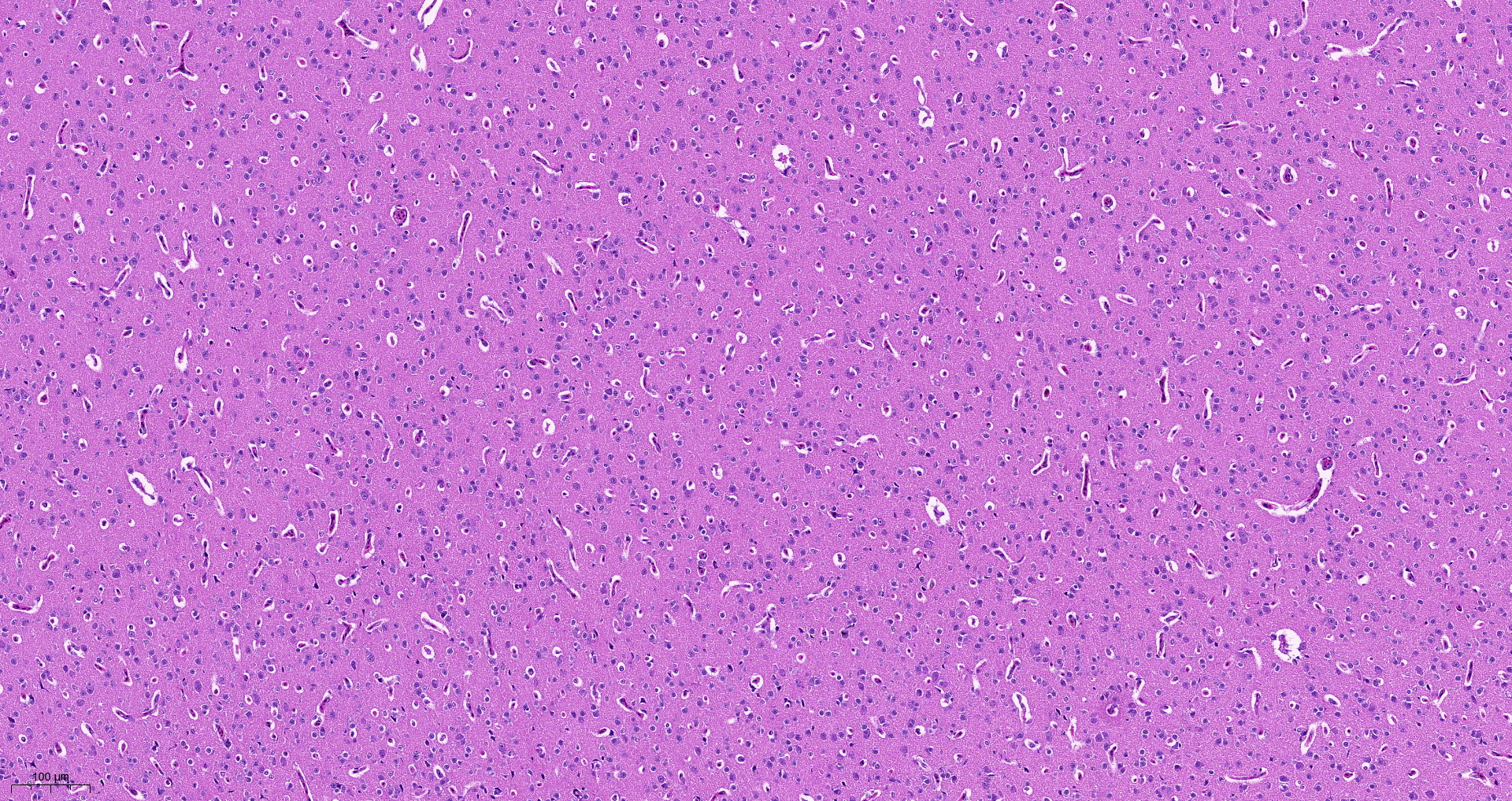

Supplement: Supplementary file 3 [file Data_Sheet_3.ZIP › duck 4dpi TMUV GX brain_10.0x.jpg]

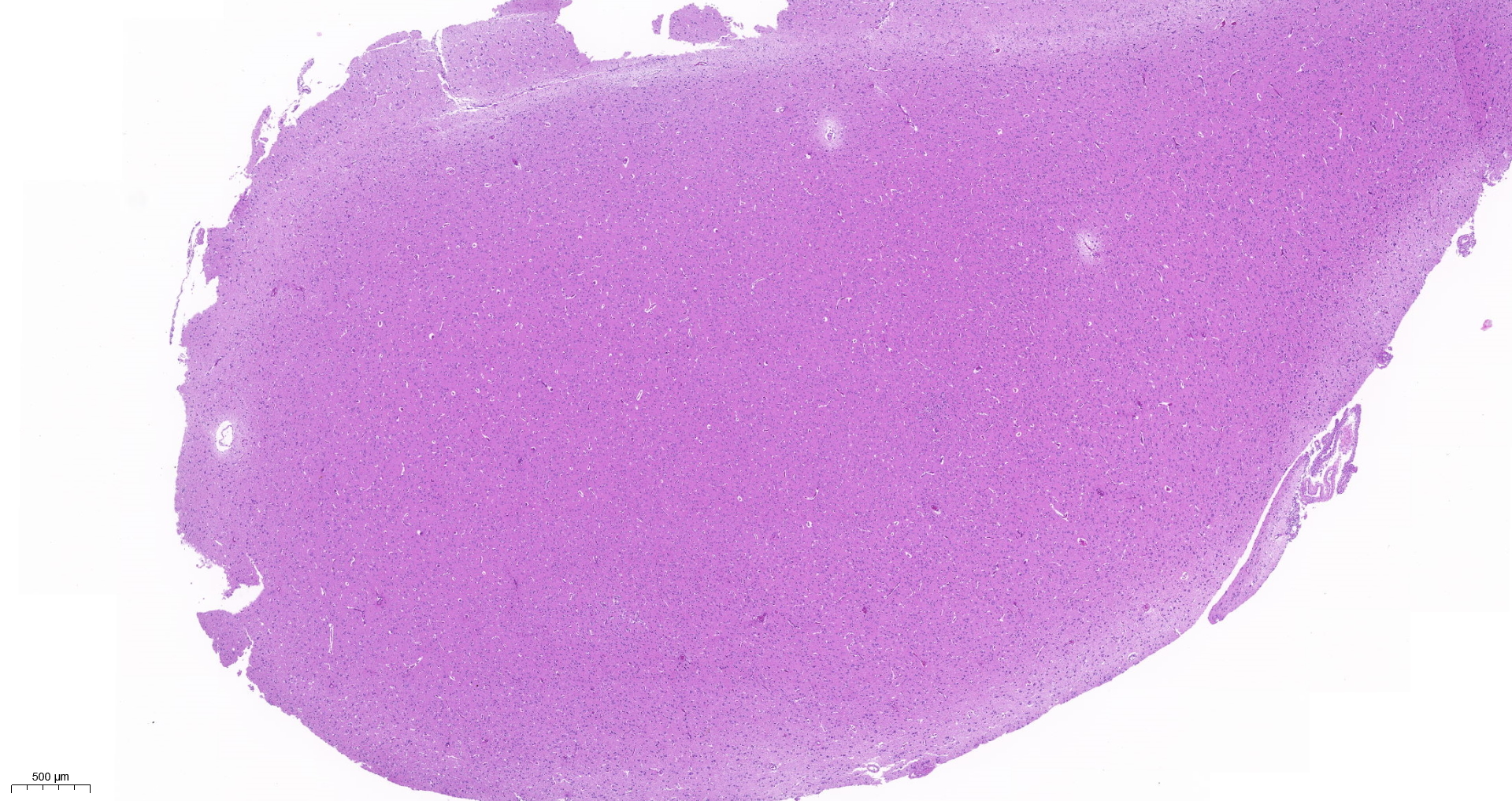

Supplement: Supplementary file 3 [file Data_Sheet_3.ZIP › duck 4dpi TMUV GX brain_2.0x.jpg]

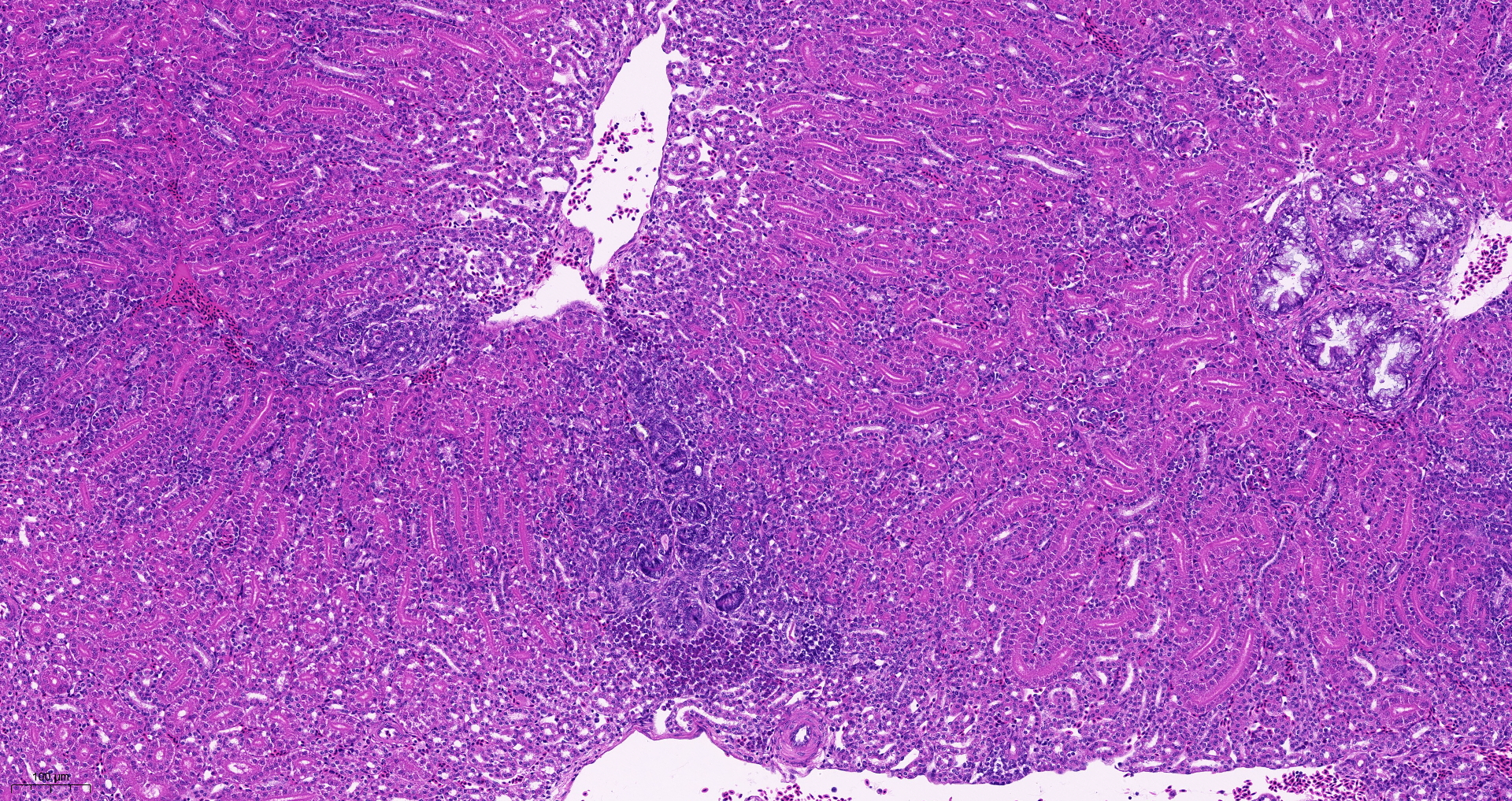

Supplement: Supplementary file 3 [file Data_Sheet_3.ZIP › duck 4dpi TMUV GX kidney_10.0x.jpg]

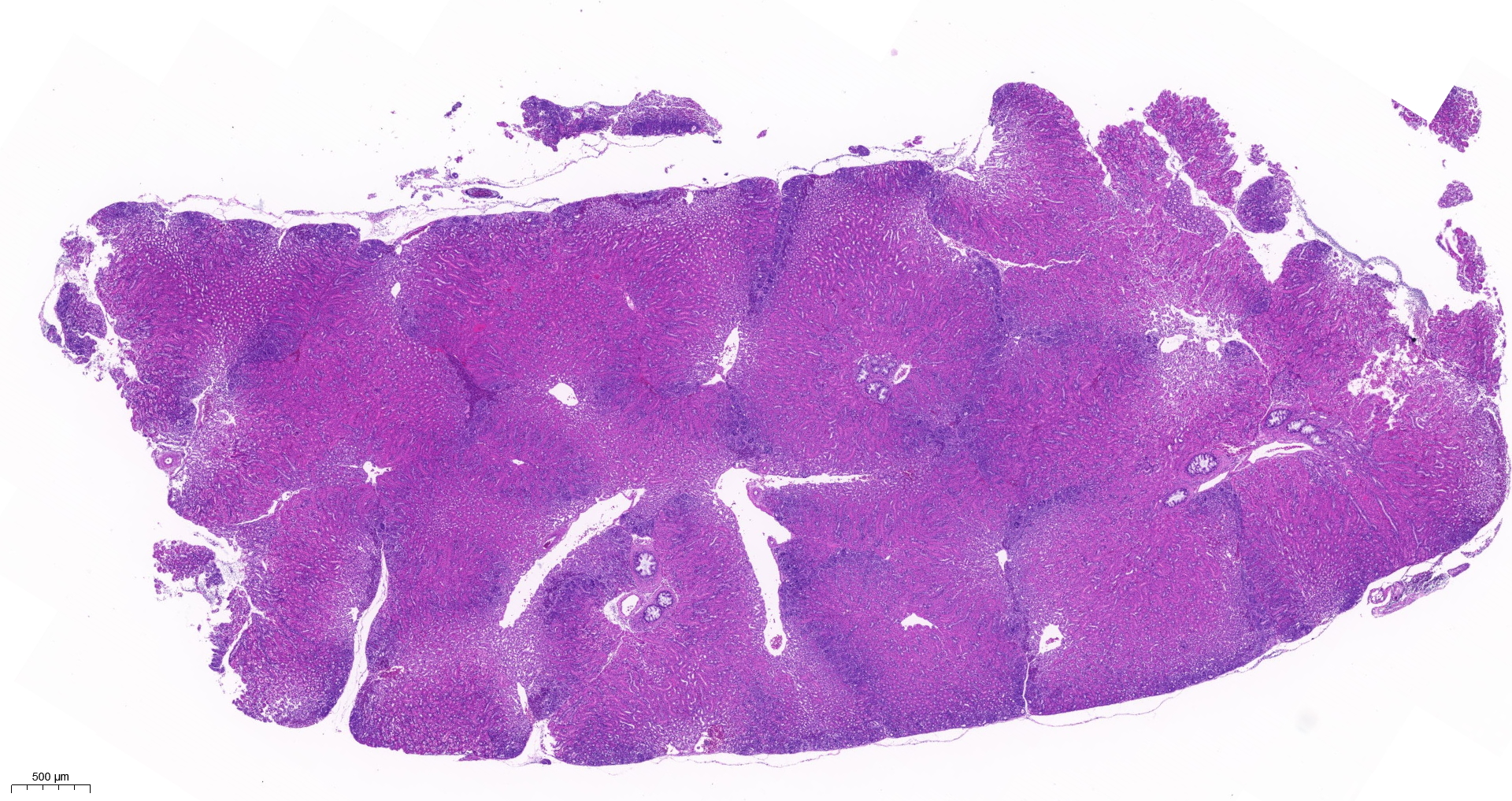

Supplement: Supplementary file 3 [file Data_Sheet_3.ZIP › duck 4dpi TMUV GX kidney_2.0x.jpg]

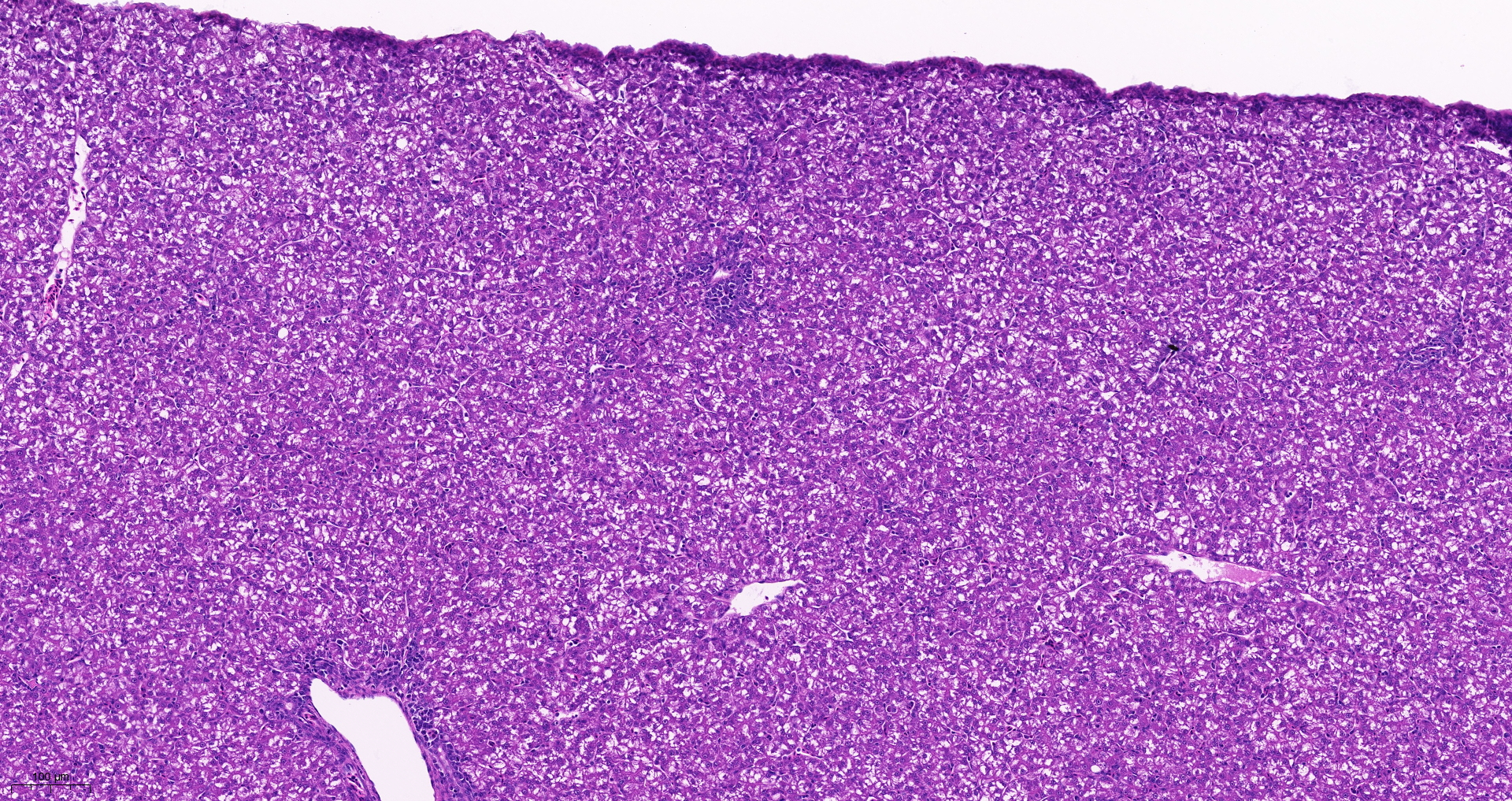

Supplement: Supplementary file 3 [file Data_Sheet_3.ZIP › duck 4dpi TMUV GX liver_10.0x.jpg]

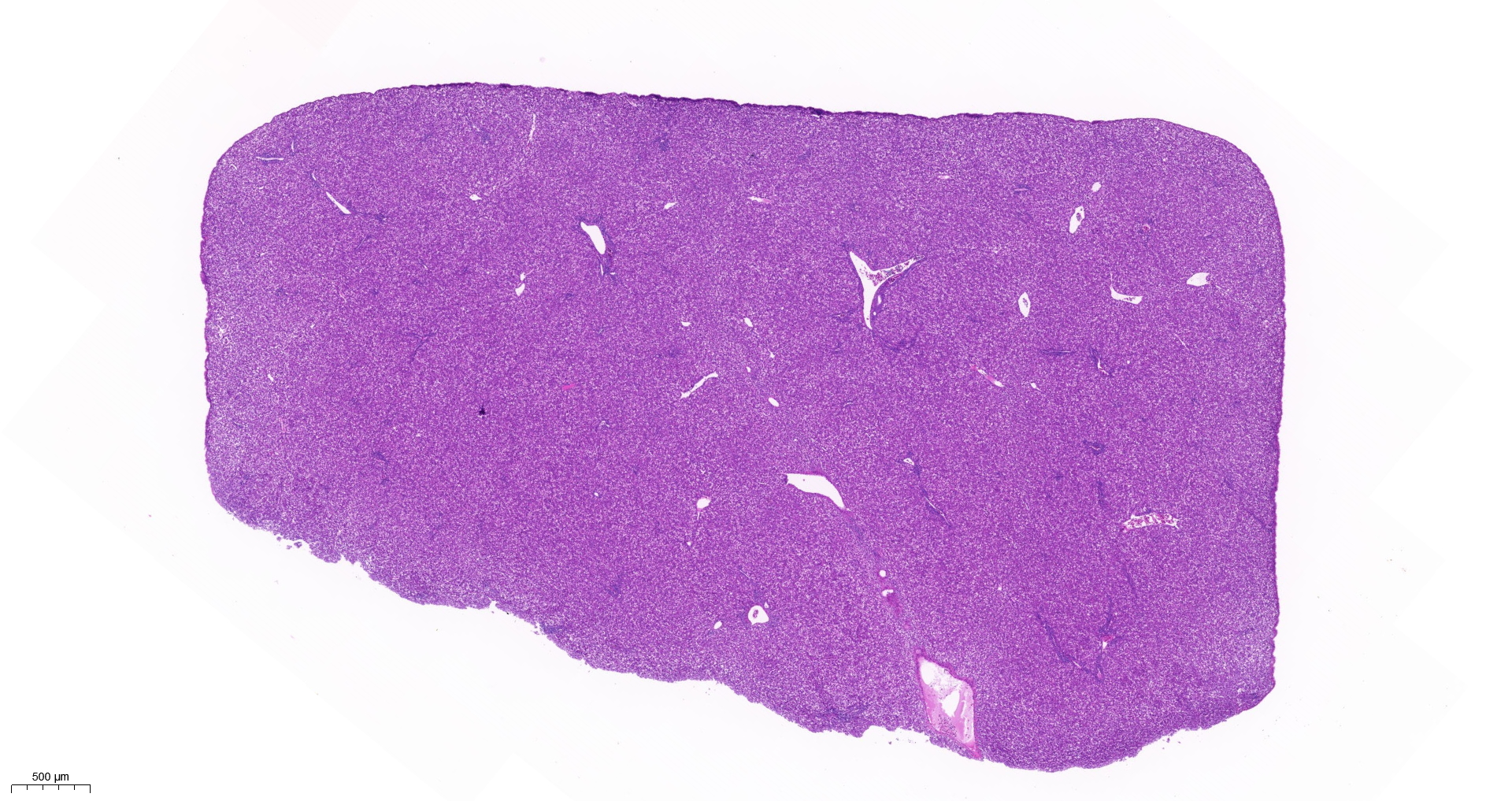

Supplement: Supplementary file 3 [file Data_Sheet_3.ZIP › duck 4dpi TMUV GX liver_2.0x.jpg]

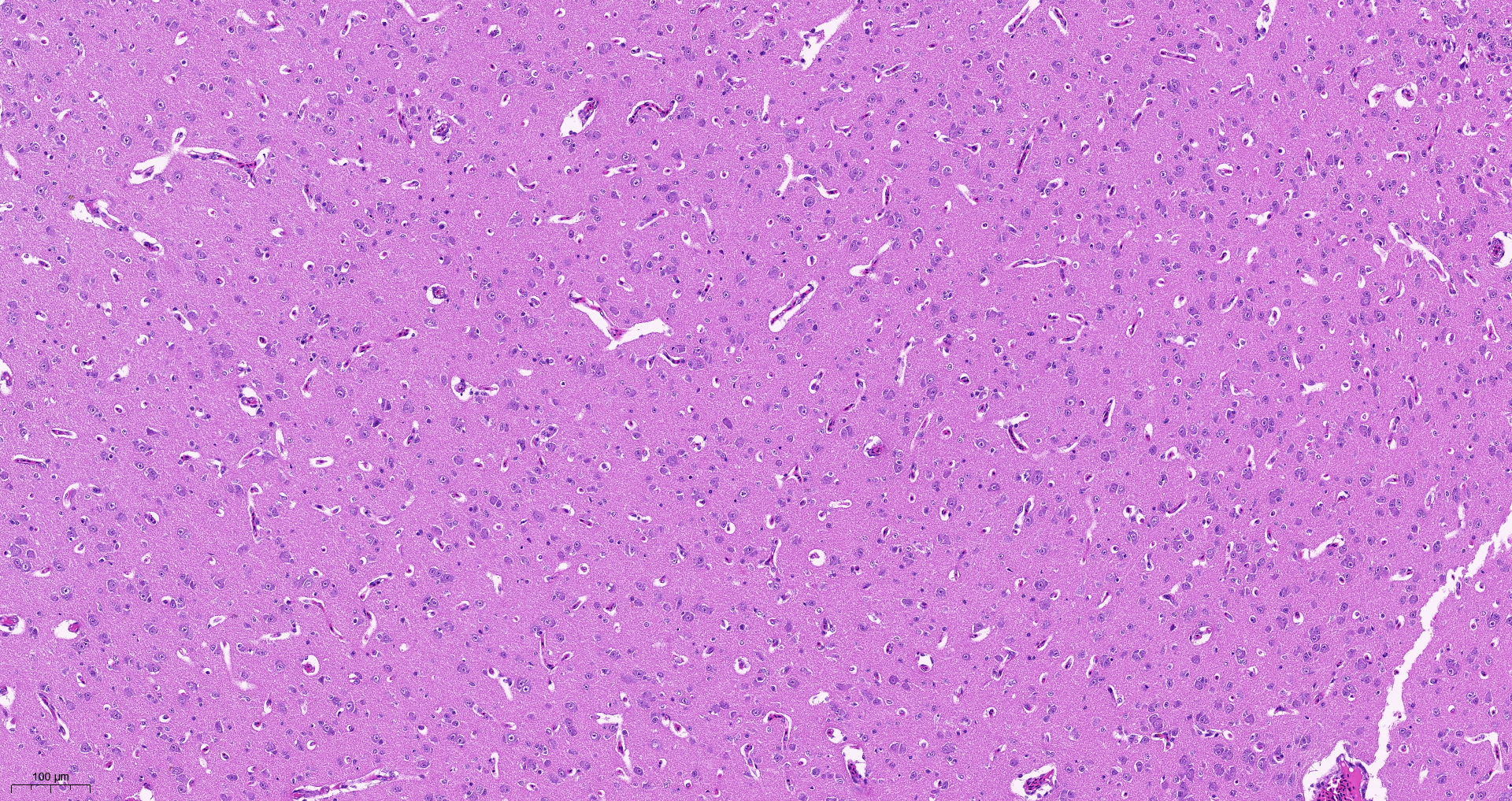

Supplement: Supplementary file 3 [file Data_Sheet_3.ZIP › duck 4dpi TMUV JM brain_10.0x.jpg]

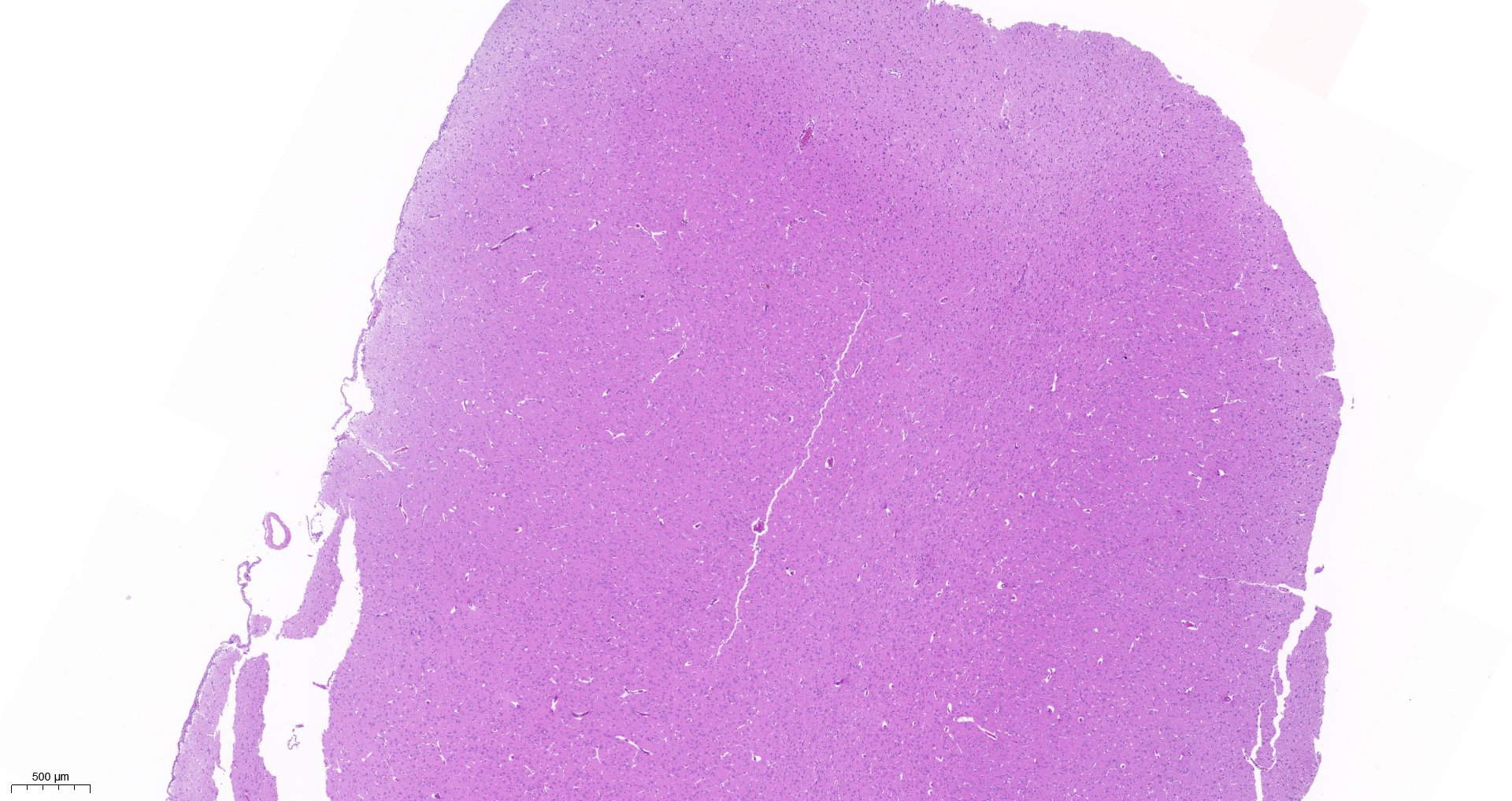

Supplement: Supplementary file 3 [file Data_Sheet_3.ZIP › duck 4dpi TMUV JM brain_2.0x.jpg]

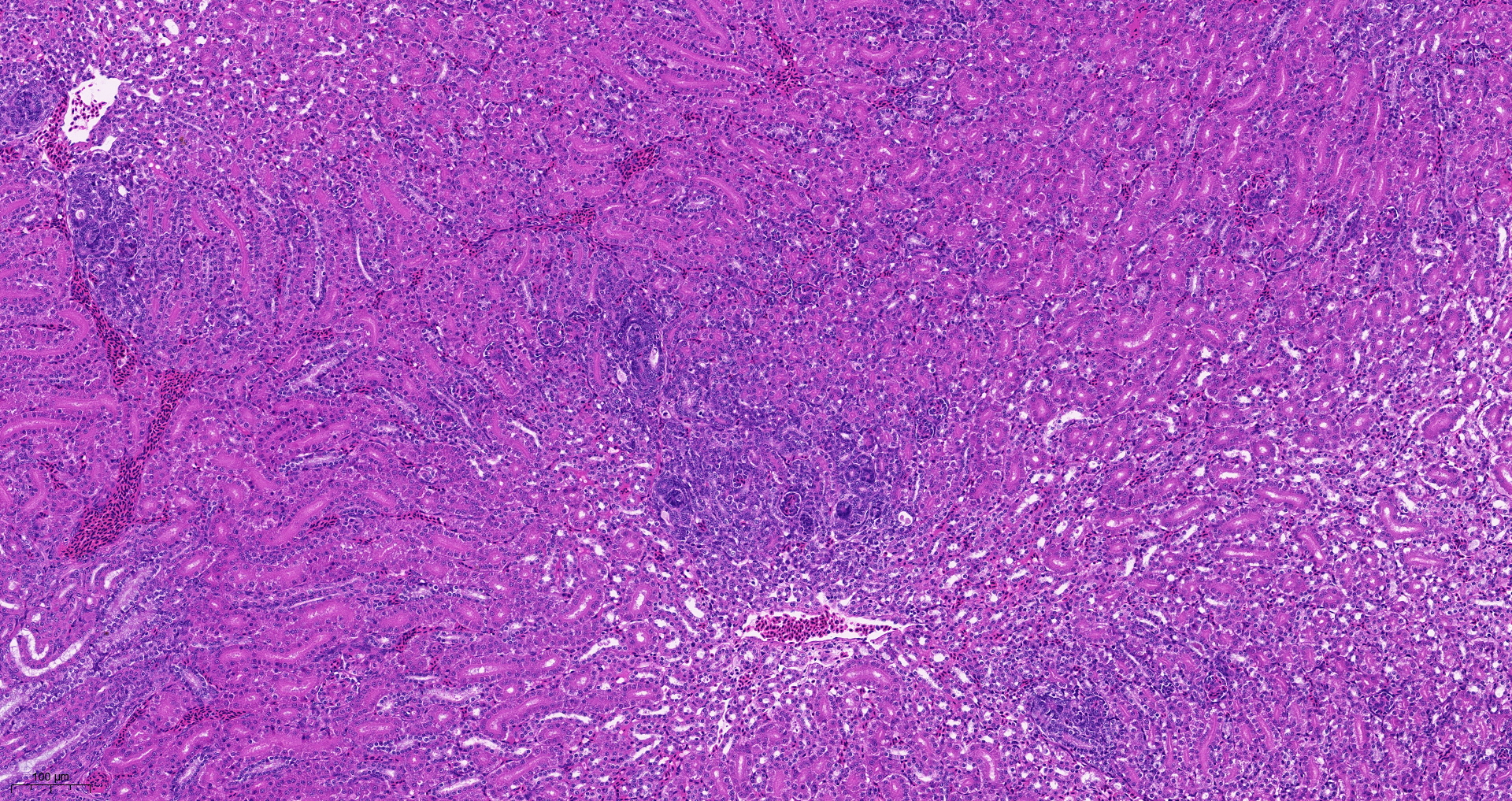

Supplement: Supplementary file 3 [file Data_Sheet_3.ZIP › duck 4dpi TMUV JM kidney_10.0x.jpg]

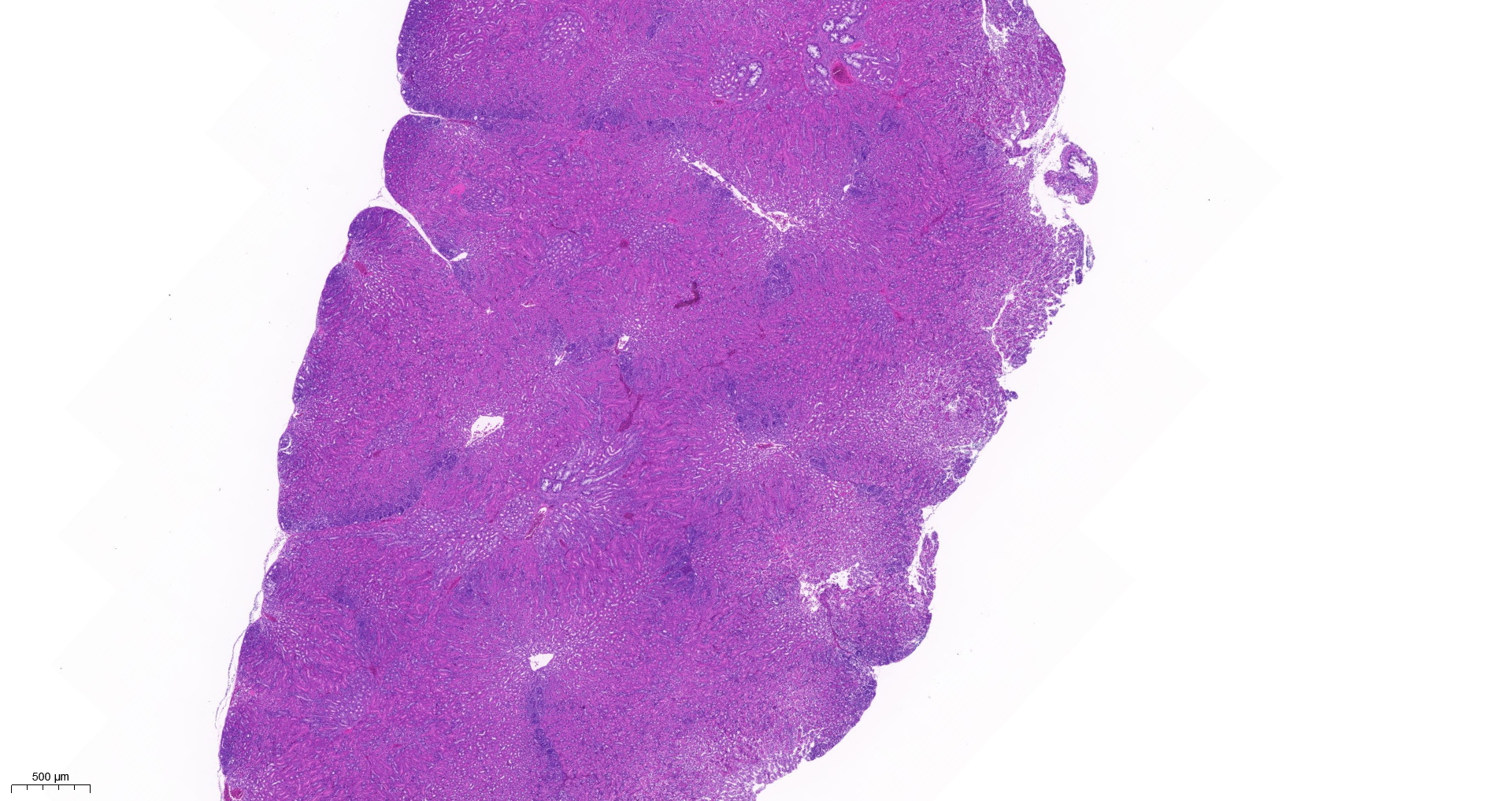

Supplement: Supplementary file 3 [file Data_Sheet_3.ZIP › duck 4dpi TMUV JM kidney_2.0x.jpg]

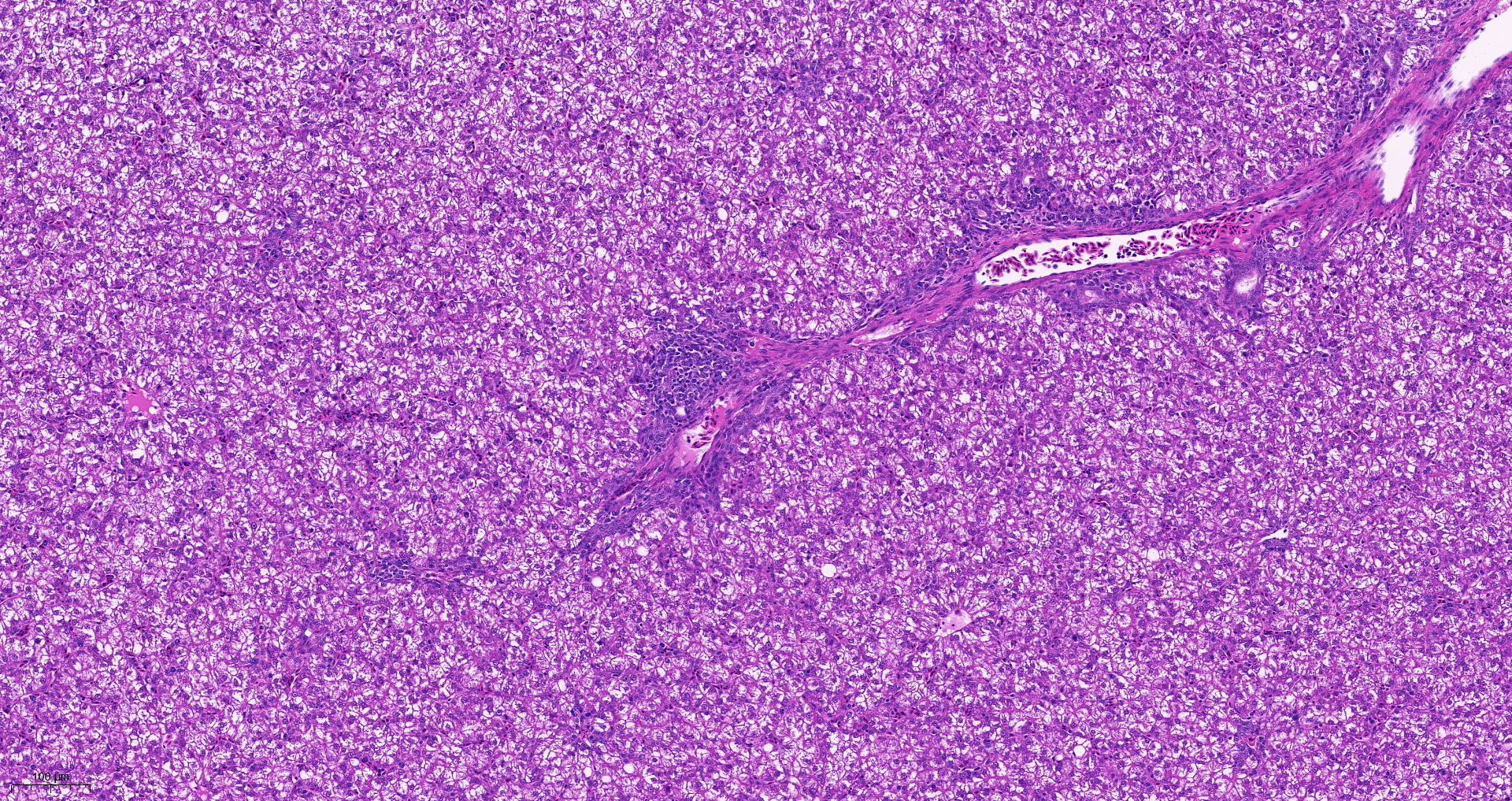

Supplement: Supplementary file 3 [file Data_Sheet_3.ZIP › duck 4dpi TMUV JM liver_10.0x.jpg]

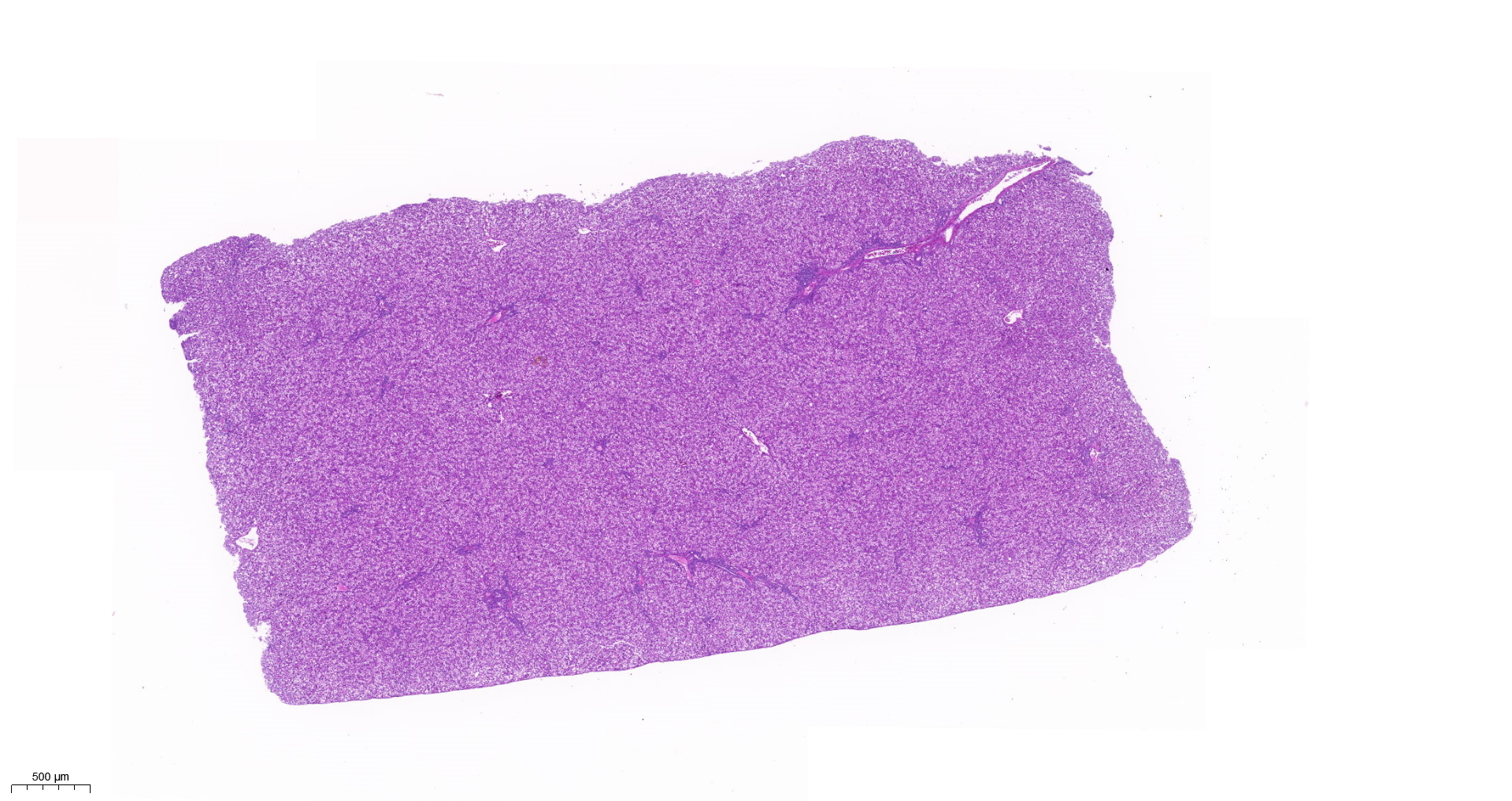

Supplement: Supplementary file 3 [file Data_Sheet_3.ZIP › duck 4dpi TMUV JM liver_2.0x.jpg]
